# Morphology, phylogeography, phylogeny, and taxonomy of *Cyclorhiza* (Apiaceae)

**DOI:** 10.3389/fpls.2024.1504734

**Published:** 2025-01-08

**Authors:** Bo-Ni Song, Xueyimu Aou, Rong-Ming Tian, Jing Cai, Wei-Yan Tan, Chang-Kun Liu, Xing-Jin He, Song-Dong Zhou

**Affiliations:** ^1^ Key Laboratory of Bio-Resources and Eco-Environment of Ministry of Education, College of Life Sciences, Sichuan University, Chengdu, China; ^2^ College of Resources Environment and Chemistry, Chuxiong Normal University, Chuxiong, China

**Keywords:** *Cyclorhiza*, morphology, phylogeography, phylogenomics, phylogeny

## Abstract

**Background:**

The genus *Cyclorhiza* is endemic to China and belongs to the Apiaceae family, which is widely distributed in the Himalaya–Hengduan Mountains (HHM) region. However, its morphology, phylogeny, phylogeography, taxonomy, and evolutionary history were not investigated due to insufficient sampling and lack of population sampling and plastome data. Additionally, we found that *Seseli purpureovaginatum* was not similar to *Seseli* members but resembled *Cyclorhiza* species in morphology, indicating that the taxonomic position of *S. purpureovaginatum* needs to be re-evaluated.

**Methods:**

First, we observed the morphology of the genus. Second, we newly sequenced four plastomes and conducted comparative analyses. Third, we used the newly sequenced internal transcribed spacer (ITS) and chloroplast DNA (cpDNA) (*mat*K, *trn*Q-*rps*16, and *trn*D-*trn*T) from 27 populations totaling 244 individuals to explore the genetic diversity and structure. Finally, we performed the phylogenetic analyses based on three datasets (plastome data, ITS sequences, and haplotypes) and estimated the origin and divergence time of the genus.

**Results and discussion:**

The morphology of *Cyclorhiza* plants and *S. purpureovaginatum* was highly similar, and their plastomes in structure and features were conserved. The genus possessed high genetic diversity and significant lineage geographic structure, which may be associated with the long-term evolutionary history, complex terrain and habitat, and its sexual reproduction mode. The genus *Cyclorhiza* originated in the late Eocene (36.03 Ma), which was closely related to the early uplift of the Qinghai–Tibetan Plateau (QTP) and Hengduan Mountains (HDM). The diversification of the genus occurred in the late Oligocene (25.43 Ma), which was largely influenced by the colonization of the newly available climate and terrain. The phylogenetic results showed that *Cyclorhiza* species clustered into a separate clade and *S. purpureovaginatum* nested within *Cyclorhiza*. *Cyclorhiza waltonii* was sister to *Cyclorhiza peucedanifolia*, and *Cyclorhiza puana* clustered with *S. purpureovaginatum*. Thus, based on the morphology, plastome analyses, and phylogenetic evidence, *S. purpureovaginatum* should be transferred to *Cyclorhiza*. All these evidences further supported the monophyly of the genus after including *S. purpureovaginatum*. Finally, we clarified the generic limits of *Cyclorhiza* and provided a species classification key index for the genus. In conclusion, the study comprehensively investigated the morphology, phylogeography, phylogeny, taxonomy, and evolution of the genus *Cyclorhiza* for the first time.

## 1 Introduction

**Figure 1:**
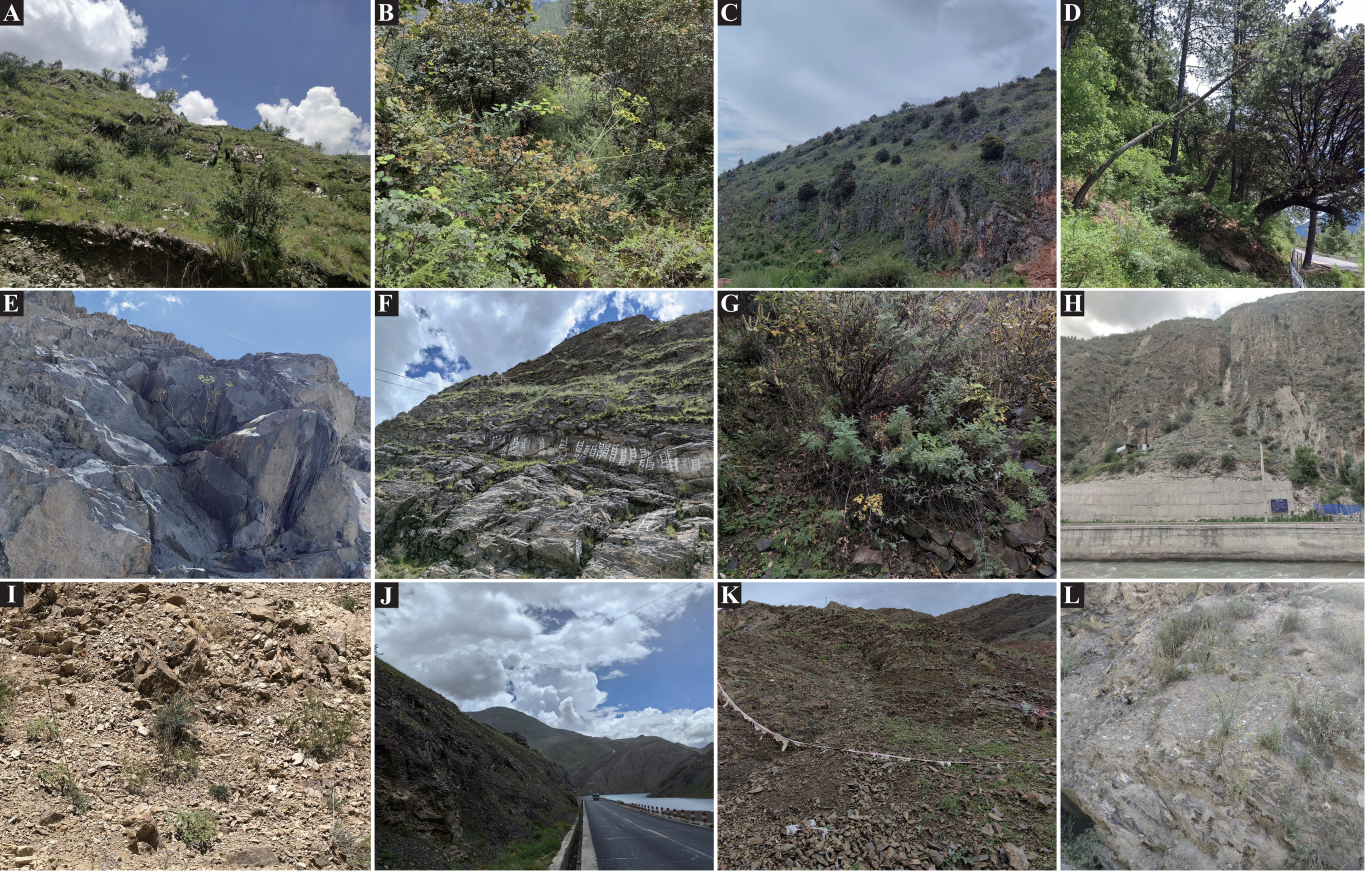
Habitats of *Cyclorhiza* members. **(A)** Sunny hillside grassland (Daocheng, Sichuan). **(B, C)** Oak forest (Minlin, Xizang; Jianchuan Yunnan). **(D)** Pine forest (Linzhi, Xizang). **(E, F)** Stone walls and crevices (Jiangzi, Xizang; Lasa, Xizang). **(G)** Scrub (Kangding Sichuan). **(H–L)** Dry rocky hillside (Derong, Sichuan; Luhuo, Sichuan; Jiangzi, Xizang; Angren, Xizang; and Xinlong, Sichuan).

**Figure 2:**
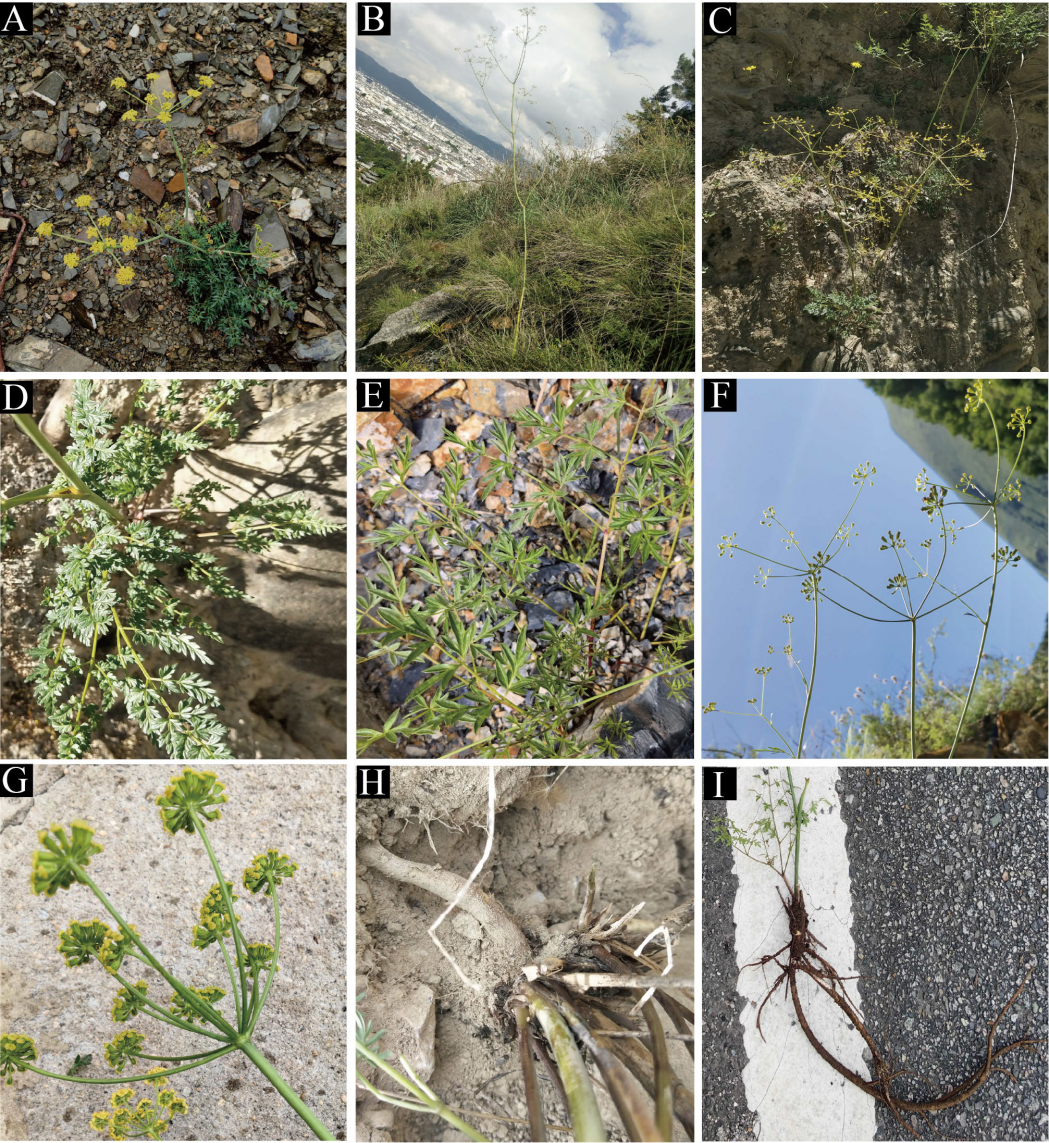
The distinctive characteristic features of *Cyclorhiza* members. **(A)** *Cyclorhiza waltonii*. **(B)** *Cyclorhiza peucedanifolia*. **(C)** *Cyclorhiza puana*. **(D, E)** Leaf. **(F, G)** Flower. **(H, I)** Root.

The genus *Cyclorhiza* (M. L. Sheh & R. H. Shan) is an endemic genus (Apiaceae) to China with high medicinal value. It was established by M. L. Sheh and R. H. Shan in 1980, with *Cyclorhiza waltonii* (H. Wolff) M. L. Sheh & R. H. Shan designated as the type species (Sheh and Shan, 1980). Its members are widely distributed in the Himalayan–Hengduan Mountains (HHM) region, which grow in open broad-leaved forests, scrublands, alpine meadows, and bamboo thickets at altitudes ranging from 1,800 to 4,600 m (Pimenov, 2017; Zhou et al., 2021) (**Figure 1**). The main morphological features of its members are characterized by carrot-like roots with prominent annular scars when old, absence of bracts/bracteoles, and laterally compressed mericarps (Sheh and Watson, 2005) (**Figure 2**).

The taxonomy of the genus is quite complex. In 1894, C. B. Clarke discovered a new species and classified it under the genus *Seseli* L., naming it as *Seseli waltonii* C. B. Clarke. However, in 1929, H. Wolff found that its morphology was similar to that of *Ligusticum* L. members and thus transferred it to the genus *Ligusticum* L., naming it *Ligusticum waltonii* (C. B. Clarke) Wolff (Wolff, 1925, 1929). In 1980, M. L. Sheh and R. H. Shan observed the topotypes of this species and found that the morphological characteristics of this species differed significantly from those of the genera *Seseli* L. and *Ligusticum* L (Sheh and Shan, 1980; Pu and Watson, 2005; Sheh and Watson, 2005; Sheh et al., 2005). For example, the mericarps commissure of this species was contracted, the seed face was deeply concave, and bracts or bracteoles were absent; in contrast, the mericarps commissure of *Seseli* L. members were not contracted, the seed face was plane, and bracteoles were numerous (Sheh and Shan, 1980; Sheh and Watson, 2005; Sheh et al., 2005). The flowers of this species were yellow with slightly laterally compressed mericarps, and mericarps were pentagonal in cross-section with vitta 1 in each furrow, whereas the flowers of the *Ligusticum* L. members were white, purple, violet, or pale pinkish, with dorsally compressed mericarps and vittae (1–)2–5 in each furrow and vittae 2–10 on commissure (Sheh and Shan, 1980; Pu and Watson, 2005; Sheh and Watson, 2005; Sheh et al., 2005). Therefore, they established a new genus, *Cyclorhiza* M. L. Sheh & R. H. Shan, to accommodate this species and designated *C. waltonii* (H. Wolff) M. L. Sheh & R. H. Shan as the type species of the genus (Sheh and Shan, 1980). Originally, the genus *Cyclorhiza* only included *C. waltonii* and a variety, *Cyclorhiza waltonii* var. *major* M. L. Sheh & Shan (Sheh and Shan, 1980). In 1997, Constance L. found that *Arracacia peucedanifolia* Franch was similar to *Cyclorhiza* members in morphology and transferred it to the genus *Cyclorhiza*, naming it as *Cyclorhiza peucedanifolia* (Franch.) Constance (1997). Later, Sheh and Watson (2005) treated *C. waltonii* var. *major* as the synonym of *C. peucedanifolia*. In 2021, Zhou et al. discovered a new species of the genus: *Cyclorhiza puana* J. Zhou & Z. W. Liu. in Luohuo (Sichuan) (Zhou et al., 2021). Thus, the genus currently comprises three species (*C. waltonii*, *C. peucedanifolia*, and *C. puana*). Interestingly, during our fieldwork, we found that *Seseli purpureovaginatum* R. H. Shan & M. L. Sheh was not similar to *Seseli* members but resembled *Cyclorhiza* members in overall morphology (**Supplementary Figure S1**). By making detailed field investigations, checking the specimen, and collecting material in type locality, we found that *S. purpureovaginatum* indeed shared some morphological features with *Cyclorhiza* members. For example, they are herbaceous and glabrous, with erect stems branching above, no bracts and bracteoles, and yellow petals. Therefore, we hypothesized that *S. purpureovaginatum* may not belong to the genus *Seseli*, and its taxonomic position needs to be re-evaluated. From the above, we noticed that the generic limits of *Cyclorhiza* and the species relationships and species identification based on morphological traits faced challenges. It is necessary to obtain more evidences to re-evaluate the generic limits of *Cyclorhiza* and further clarify their interspecific boundaries.

Previous studies have confirmed that the fruit and pollen characteristics were the two most important morphological characteristics in the classification system of the Apiaceae (Song et al., 2024a). They have been widely used in taxonomic studies of many notorious genera of Apiaceae, such as *Sanicula* L. (Song et al., 2024a), *Angelica* L. (Yang and Liao, 2023), *Bupleurum* L. (Zhou et al., 2018), *Peucedanum* L. (Zhang and He, 2009), and *Cnidium* Cusson (Li et al., 1993). However, the morphological and micromorphological studies of fruits and pollens on the genus *Cyclorhiza* have not been performed comprehensively, and only three micromorphological studies have been reported so far (Sheh and Shan, 1980; Shu and Sheh, 2001; Zhou et al., 2021). Namely, Sheh & Shan observed and described the fruit morphology of *C. waltonii* and *C. peucedanifolia* in 1980 (Sheh and Shan, 1980). In 2001, Shu and Sheh observed the micromorphological features of pollens of *C. waltonii* and *C. peucedanifolia* (Shu and Sheh, 2001). Zhou et al. (2021) discovered a new species (*C. puana*) and described its main morphological characteristics, including the fruits’ features. Although these morphological and micromorphological studies have greatly filled the gaps and provided valuable references for the genus *Cyclorhiza*, they only described the fruit and pollen features of some species. In addition, previous studies have not performed a comprehensive morphological/micromorphological comparison of fruits and pollens for all *Cyclorhiza* species, and the value of morphological/micromorphological studies for the taxonomy and evolution of this genus was also unclear. Therefore, it is urgent to further investigate the morphological/micromorphological features of this genus based on expanding sampling.

The HHM region, one of Earth’s 34 biodiversity hotspots, is characterized by its unique geology and dramatic topography (Sun et al., 2017). It is also considered to be the diversification center of many organisms and acted as the primary source area for dispersal to many areas of the world during the last 30 million years (Donoghue and Smith, 2004). The Himalayas define the southern margin of the QTP, whereas the HHM in southwest China forms the southeastern boundary of the plateau (Zhang et al., 2002). Therefore, the HHM region was regarded as the largest evolutionary front of the North Temperate Zone (Wu, 1988; Hughes and Atchison, 2015). Previous studies mainly focused on the species-level diversification caused by the uplift of the QTP, with few studies specifically devoted to an endemic genus of the HHM region (Liu et al., 2006; Wang et al., 2009; Xia et al., 2022). The genus *Cyclorhiza* is endemic to HHM region (Zhou et al., 2021); thus, it is an ideal object for studying how species genetic diversity responds to historical events of regional environmental change. However, no systematic biogeographic and phylogenetic study on the genus *Cyclorhiza* has been conducted, nor has the evolutionary history of *Cyclorhiza* been studied. Therefore, we tried to explore the genetic distribution pattern, genetic diversity and structure, origin, and diversification of the genus from phylogenetic and phylogeographic analyses.

With the advancements in next-generation sequencing (NGS) and bioinformatics technologies, plastome data have been widely and successfully used to generate high supports and resolutions of plant phylogenies at order, family, and genus levels, such as in the order Saxifragales Bercht. & J. Presl (Jia et al., 2024), family Apiaceae (Wen et al., 2021; Liu et al., 2022; Ren et al., 2022; Gui et al., 2023; Qin et al., 2023; Song et al., 2023; Chen et al., 2024; Guo et al., 2024; Song et al., 2024b, c), Loranthaceae Juss (Tang et al., 2024), Liliaceae (Zhou et al., 2024a), subfamily Nolinoideae (Ji et al., 2023), genus *Paris* L (Zhou et al., 2024b), and *Polygonatum* Mill (Wang et al., 2023). However, there is no phylogenetic framework related to the genus *Cyclorhiza* based on plastome data yet. Previous molecular studies related to the *Cyclorhiza* mainly used single- or multiple-locus DNA sequence data, such as ITS sequence cpDNA (*rpl*16 and *rps*16 intron), plastid DNA *rpl*16 and *rps*16 intron (Downie et al., 2000; Zhou et al., 2009), yet these studies failed to provide sufficient information to support the improvement of taxonomy for *Cyclorhiza* due to involved limited *Cyclorhiza* species. Therefore, in order to comprehensively understand the phylogeny, phylogeography, phylogenomics, and taxonomy of the genus, we used plastome data in this current study. Our major aims were to 1) compare the morphological/micromorphological features of the *Cyclorhiza* members; 2) characterize the plastomes of *Cyclorhiza* plants and select highly variable hotspot regions as candidate DNA barcodes for species authentication of the genus; 3) investigate the genetic diversity and genetic structure, origin, and diversification of the genus based on materials from 244 individuals from 27 populations; and 4) reconstruct the phylogenetic framework of the genus based on three datasets (ITS sequence, plastome data, and haplotype) and clarify the generic limits of the genus *Cyclorhiza* and the interspecific relationships within the genus.

## 2 Materials and methods

### 2.1 Plant materials and morphological observations

**Figure 3:**
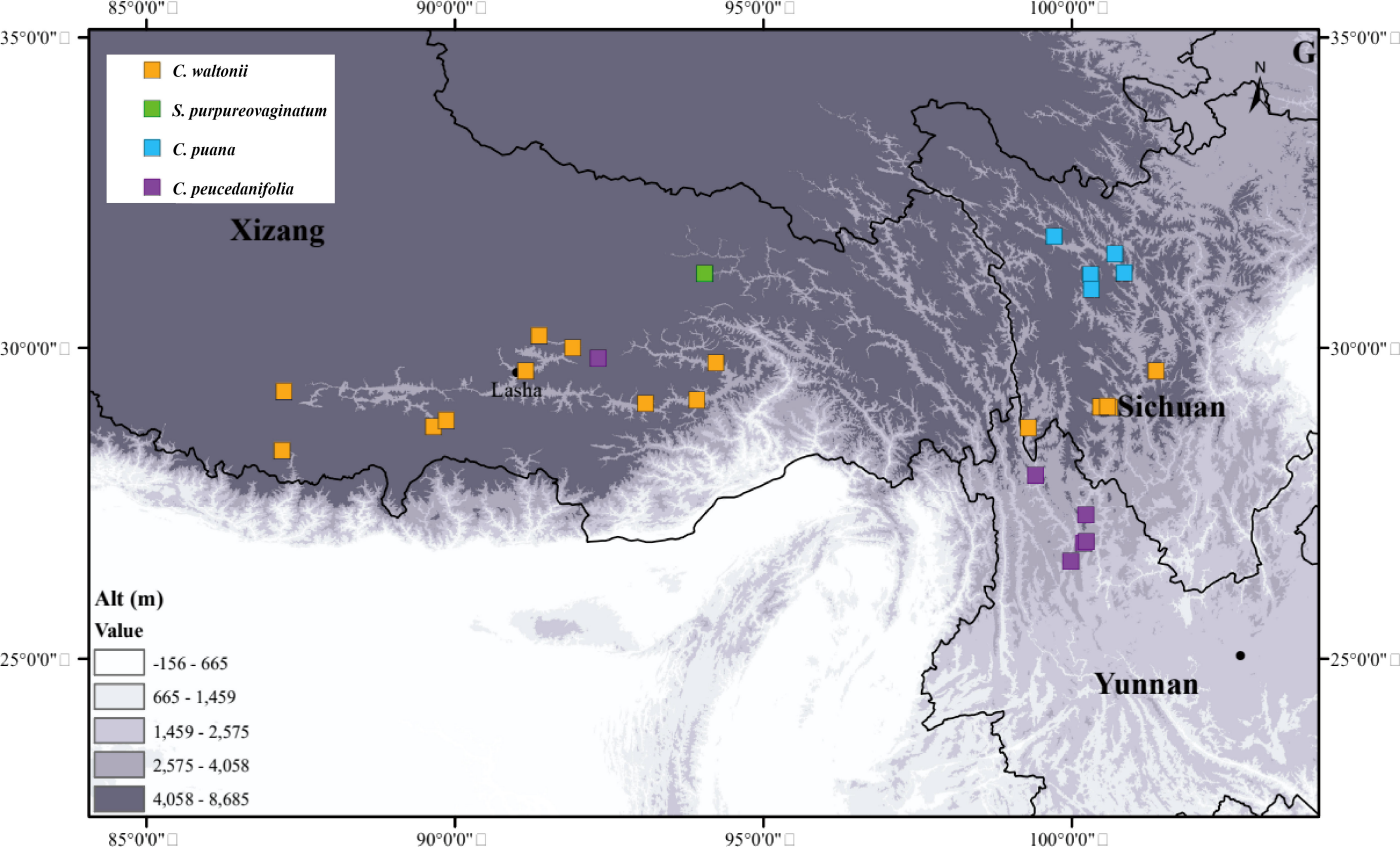
Population sampling distribution points involved in this study.

We collected 244 individuals from 27 populations of four species between 2021 and 2024. These individuals grow in western Sichuan, northwestern Yunnan, and southern Xizang, most of them with altitudes above 3,000 m (**Figure 3**; **Supplementary Table S1**). The collection of each species covered the type locality and the entire distribution area, and we randomly collected five to fifteen individuals from each population. We immediately stored the fresh young basal leaves and dried them with silica gel. We deposited the voucher specimens in the herbarium of Sichuan University (Chengdu, China, SZ) (**Supplementary Table S1**).

In addition, we also collected their mature fruits and pollens in the field to observe their micromorphological characteristics. First, we selected 30 representative fruit samples of each species to observe their overall structure and anatomy using a stereo microscope (SMZ25, Nikon Corp., Tokyo, Japan). Second, we selected 10 dry, mature, and full anthers of each species and pasted them on the double-sided conductive adhesive; we gently poked the pollen capsule with a clean dissecting needle to release the pollens, coated them using gold spray, and used a JSM-7500F scanning electron microscope to observe and photograph the overall view of pollen, the equatorial view, the polar view, germ furrow, and the exine ornamentation. We used the software MATO (Liu et al., 2023) to measure the 30 representative fruit samples and 10 pollens for each species, and then we calculated the average value. The description of fruit and pollen characteristics referred to the studies of Kljuykov et al. (2004), Ostroumova (2021), and Shu and Sheh (2001). Furthermore, we also obtained other morphological characteristics of *Cyclorhiza* members based on extensive documentation, specimen information, and fieldwork.

### 2.2 DNA extraction, amplification, sequencing, assembly, and annotation

The total genomic DNA of newly collected samples was extracted from silica gel-dried leaves using the modified cetyltrimethylammonium bromide (CTAB) method (Doyle and Doyle, 1987). The quality and quantity of genomic DNA were tested via 1% agarose gel electrophoresis. For plastome data, the DNA library with an average insert size of 300–400 bp was constructed using the TruSeq DNA Sample Preparation Kits (Illumina) referred to the manufacturer’s protocol (Illumina, San Diego, CA, USA). The DNA library was sequenced using the Illumina HiSeq2500 platform at Novogene (Beijing, China), applying the paired-end 150-bp reads. Then, the software fastP v0.15.0 (-n 10, -q 15) (Chen et al., 2018) was used to filter the raw data and yield clean data (at least 5 GB). For the yielded clean data, the GetOrganelle pipeline (Jin et al., 2020) was used to assemble the complete plastomes, setting the plastome sequence of *Changium smyrnioides* H. Wolff (MN092718) as a reference. The complete plastomes were initially annotated by the Plastid Genome Annotator (PGA) software (Qu et al., 2019), with *C. smyrnioides* H. Wolff (MN092718) as a reference, and then the start and stop codons and intron positions were manually checked and corrected in Geneious v9.0.2 (Kearse et al., 2012). Finally, the well-annotated plastomes were displayed using the online program OrganellarGenomeDRAW (OGDRAW) (Stephan et al., 2019).

For the ITS region, the sequence of 244 individuals from 27 populations was amplified using 30-µL polymerase chain reaction (PCR) system, including 2 µL total DNA, 1.5 µL forward primers ITS-4 (5′-TCCTCCGCTTATTGATATGC-3′), 1.5 µL reverse primers ITS-5 (5′-GGAAGTAAAAGTCGTAACAAGG-3′), 15 µL Taq MasterMix (CWBio, Beijing, China), and 10 µL of ddH_2_O. The PCR amplification program was as follows: 4 minutes of initial denaturation at 94°C; 36 cycles consisting of denaturation at 94°C for 45 seconds, annealing at 52°C for 70 seconds, extension at 72°C for 90 seconds, a final extension at 72°C for 10 minutes; and stored at 4°C freezer (White, 1990). Finally, the PCR products were sent to Sangon (Shanghai, China) for sequencing. The newly generated 244 ITS sequences were assembled and edited using the software Geneious v9.0.2 (Kearse et al., 2012) to gain the consensus sequences.

For cpDNA fragments, we first amplified and sequenced 10 cpDNA regions (*rpl*16, *rpl*32–*trn*L, *trn*D–*trn*T, *trn*H–*psb*A, *trn*Q–*rps*16, *trn*S–*trn*G, *rbc*L, *rps*16–*trn*K, *mat*K, and *rps*16) of 27 individuals from 27 populations to screen out cpDNA fragments that exhibited significant differences at the population level. The primer sequences for these 10 regions are provided in the **Supplementary Table S2**. Finally, we selected three cpDNA fragments (*mat*K, *trn*Q–*rps*16, and *trn*D–*trn*T) and amplified these three fragments of all individuals. The PCR amplification system for each cpDNA fragment was 30 µL, including 2 µL total DNA, 1.5 µL forward primers and 1.5 µL reverse primers, 15 µL Taq MasterMix (CWBio, Beijing, China), and 10 µL of ddH_2_O. The PCR amplification program was as follows: initial denaturation at 94°C for 4 minutes, followed by 35 cycles of denaturation at 94°C for 45 seconds, annealing at 52°C for 45 seconds, extension at 72°C for 1 minute, and a final extension step at 72°C for 7 minutes. Finally, we sent the amplified products to Sangon (Shanghai, China) for bidirectional sequencing. We used the software Geneious v9.0.2 (Kearse et al., 2012) to assemble and edit the newly generated cpDNA fragment sequences and gain the consensus sequences.

All new plastome data, ITS sequences, and cpDNA fragments were submitted to the National Center for Biotechnology Information (NCBI), and the accession numbers were shown in **Supplementary Table S3**.

### 2.3 Plastome comparison and hotspots identification

First, four types of repeat sequences (forward, reverse, complement, and palindromic) were detected using the online program REPuter. The parameters were as follows: maximum computed repeats >90%, minimal repeat size ≥30 bp, and a hamming distance = 3 (Kurtz et al., 2001). The simple sequence repeats (SSRs) were also analyzed using the Perl script MISA (https://webblast.ipk-gatersleben.de/misa/), and the minimum number of repeat units parameter was set to ten repetitions for mononucleotides, five repetitions for dinucleotides, four repetitions for trinucleotides, and three repetitions for tetranucleotides, pentanucleotides, and hexanucleotides (Beier et al., 2017). Second, the boundaries between inverted repeat and single-copy (IR/SC) were visualized using the online tool IRscope (https://irscope.shinyapps.io/irapp) (Amiryousef et al., 2018) after manual adjustment. Third, the sequence divergence of whole plastomes was detected and visualized using the online program mVISTA viewer in Shuffle-LAGAN mode (Frazer et al., 2004), with *C. waltonii* as a reference. Fourth, the DNA rearrangements were detected using Mauve Alignment (Darling et al., 2004) implemented in Geneious v9.0.2 (Kearse et al., 2012), with other parameters set as the default values. Fifth, the CodonW v1.4.2 program (Peden, 1999) was employed to compute the relative synonymous codon usage (RSCU) (Sharp and Li, 1986) of these shared coding sequences (CDSs) (>300 bp). Finally, DNAsp v5.0 (Librado and Rozas, 2009) was used to calculate the nucleotide diversity values (Pi).

### 2.4 Genetic diversity and structure

First, we used DNAsp v5.0 (Librado and Rozas, 2009) to calculate and count the haplotype types and numbers, and we subsequently estimated the nucleotide diversity (π) (Nei and Li, 1979) and haplotype diversity (*H*d) (Nei and Tajima, 1981) of each population and the whole population. Second, we employed PERMUT (Pons and Petit, 1996) to compute the total diversity (*H*_T_), within-population diversity (*H*_S_), and population differentiation indices (*G*_ST_ and *N*_ST_) (Grivet and Petit, 2002). We also used U-statistics to compare *G*_ST_ and *N*_ST_ values between populations. Additionally, we performed analyses of molecular variance (AMOVA) with 1,000 permutations using ARLEQUIN v3.5 (Excoffier and Lischer, 2010) to detect the genetic variation among species F_SC_, among populations within species F_ST_, and within population F_CT_. Finally, we used the PopART 1.7 software (Polzin and Daneshmand, 2003) to construct the network of ITS haplotypes and cpDNA haplotypes.

### 2.5 Phylogenetic analyses and divergence time estimation

To better investigate the phylogeny of the genus *Cyclorhiza*, the phylogenetic trees were reconstructed based on three datasets: dataset 1 was the 39 complete plastomes, dataset 2 was 92 ITS sequences, and dataset 3 was haplotypes (28 ITS haplotypes and 35 cpDNA haplotypes) (**Supplementary Table S4**). Among them, the *Bupleurum* L. species were chosen as the outgroup referred to the previous research (Zhou et al., 2021). The ITS sequences and haplotype sequences were straightway aligned with MAFFT v7.221 (Katoh and Standley, 2013) to gain the matrix. For plastome data, 79 commonly shared protein coding sequences (CDSs) of 39 species were manually extracted in Geneious v9.0.2 (Kearse et al., 2012) and aligned with MAFFT v7.221 (Katoh and Standley, 2013). Then, the alignments were trimmed using trimAI (Capella-Gutierrez et al., 2009) and finally concatenated as a super matrix using PhyloSuite v1.2.2 (Zhang et al., 2020). Two methods [maximum likelihood (ML) and Bayesian inference (BI)] were employed to construct the phylogenetic trees. For the ML method, RAxML v8.2.8 (Stamatakis, 2014) with the GTRGAMMA model and 1,000 bootstrap replicates was suggested to estimate the support value [bootstrap value (BS)] for each node referred to in the RAxML manual. For the BI method, MrBayes v3.2.7 (Ronquist et al., 2012) was utilized for Bayesian inference, and the best-fit nucleotide substitution model (GTR + F + I + G4) for the matrix of the CDS dataset, (SYM + G4) for the ITS dataset, (GTR + G) for ITS haplotype, and (GTR + I + G) for cpDNA haplotype were determined using Modeltest v3.7 (Posada and Crandall, 1998). Two independent Markov chain Monte Carlo (MCMC) runs of 10 million generations were performed with sampling every 1,000 generations. The MCMC runs finished when the average standard deviation of the splitting frequency fell below 0.01. The initial 25% of trees were discarded as burn-in, and the remaining trees were used to generate the consensus trees and calculate posterior probabilities (PP). Finally, all results of ML and BI phylogenetic analyses were visualized and edited using FigTree v1.4.2 (Rambaut and Drummond, 2015).

To explore the origin and diversification of the genus, the phylogenetic tree of ITS haplotypes was selected to estimate the divergence time. Bayesian relaxed clock analysis in the program Bayesian Evolutionary Analysis Sampling Trees (BEAST v1.10.4) (Suchard et al., 2018) was performed. Pollen calibration points of *Bupleurum*, as referred to in previous studies (Calvino et al., 2016; Wen et al., 2021), were selected to constrain the phylogenetic tree, lognormal distribution was applied, and the set offset value was 33.9 and the mean value was 2.389, as referred to in published studies (Banasiak et al., 2013; Wen et al., 2021). BEAUti was employed to set criteria under the uncorrelated relaxed molecular clock model and a Yule tree prior. Modeltest v3.7 (Posada and Crandall, 1998) was used to detect the best-fit nucleotide substitution model (GTR + G). MCMC analysis was run for 10 million generations with parameters sampled every 10,000 generations after discarding the first 20% of generations as burn-in. The convergence of the stationary distribution was accessed by effective sample size (ESS) values (>200) using the software Tracer v.1.7.1 (Rambaut et al., 2018). Maximum clade credibility (MCC) tree was produced using the TreeAnotator v2.1.2 software (Rambaut and Drummond, 2014), and the result was visualized using FigTree v1.4.42 (Rambaut and Drummond, 2015) and Interactive Tree Of Life (iTOL) (Letunic and Bork, 2007).

## 3 Results

### 3.1 The morphological and micromorphological analyses

**Figure 4:**
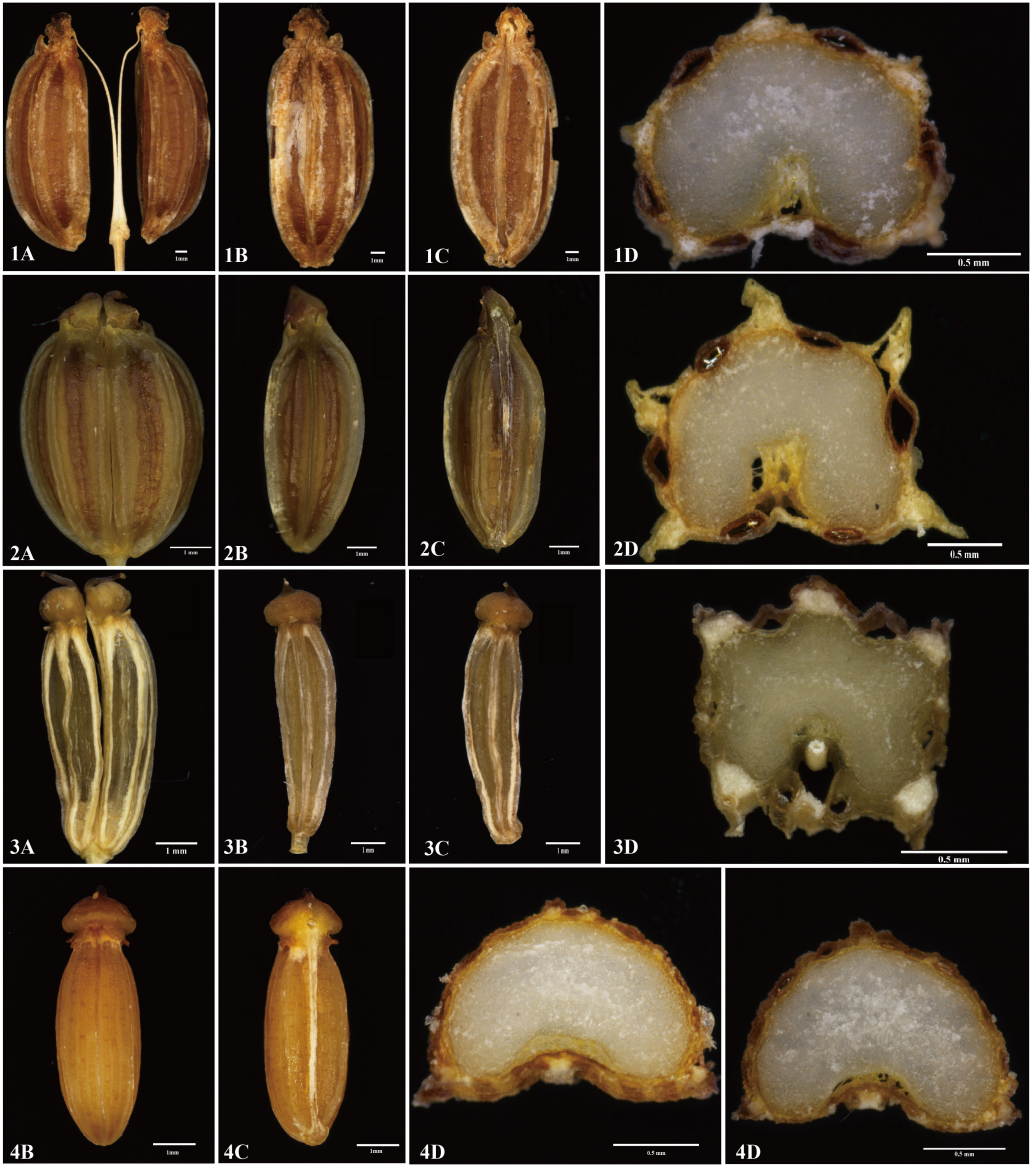
The fruits’ features of four members. 1: *Cyclorhiza waltonii*. 2: *Cyclorhiza peucedanifolia*. 3: *Cyclorhiza puana*. 4: *Seseli purpureovaginatum.* **(A)** Cremocarp. **(B)** Dorsal view. **(C)** Commissural side view. **(D)** Transverse section view. Scale bar=1mm **(A–C)**; Scale bar=0.5mm **(D)**.

**Figure 5:**
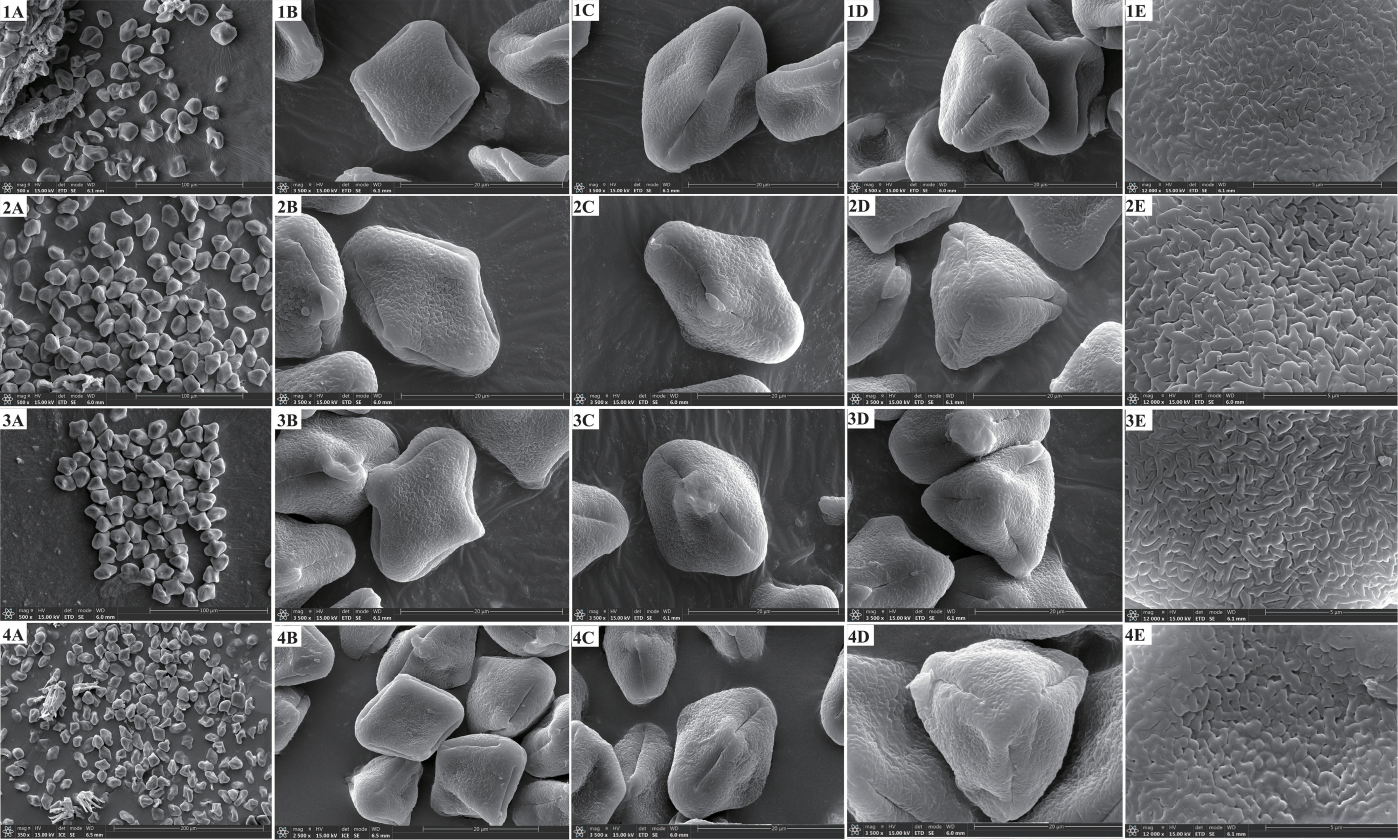
The pollen features of four members. 1: *Cyclorhiza waltonii*. 2: *Cyclorhiza peucedanifolia*. 3: *Cyclorhiza puana*. 4: *Seseli purpureovaginatum*. **(A)** Pollen grain. **(B)** Equatorial view. **(C)** Germ furrow. **(D)** Polar view. **(E)** Exine ornamentation.

We found that the key external morphological characteristics of *Cyclorhiza* members were highly similar, such as being herbaceous and glabrous, carrot-like roots with prominent annular scars when old, stem fistulose, erect, branched above, base clothed in purplish-brown remnant sheaths, absence bracts, and bracteoles and with yellow petals (**Figure 2**; **Table 1**). In addition, we also observed the fruit micromorphological features (fruit appearance, fruit size, and anatomical characteristics) of the genus and detected that their fruit shapes were ovoid or ellipsoid, smooth and glabrous, slightly laterally compressed, mericarps subpentagonal in cross-section, ribs 5, filiform, prominent, acute-ridged, almost narrowly winged, vittae 1–2 in each furrow and 2–4 on commissure, with the endosperm commissural face deeply sulcate or concave (**Figure 4**). However, we observed some differences among them, such as the fruit size ranging from (3–5.5) × (1–2.5) mm to (4–7) × (2–3.5) mm (**Table 1**). Moreover, the pollen of *Cyclorhiza* species also shared some similarities, such as the equatorial view was rombiformis, the polar view was subtriangular and had an angular germinal aperture, and the exine ornamentation of the equatorial view was short rod-like pseudo-cerebroid (**Figure 5**). However, the pollen size was different, the polar axis was 24.52–34.63 μm, the equatorial axis was 18.84–25.65 μm, and the ratio of the polar axis to the equatorial axis was 1.12–1.35 μm (**Table 1**). Furthermore, we found that the external morphological and micromorphological features (fruit and pollen) of *S. purpureovaginatum* were very similar to those of *Cyclorhiza* plants (**Supplementary Figures S1**; **Figures 2**, **4**, **5**; **Table 1**).

**Table 1:** The morphological and micromorphological characteristics of four plants.

	Taxa	*Cyclorhiza waltonii*	*Cyclorhiza peucedanifolia*	*Cyclorhiza puana*	*Seseli purpureovaginatum*
Overall morphology	Root	Annular scars, slender	Annular scars, stout	Annular scars, stout	Annular scars, stout
	Stem	Simple	Simple	Simple	Simple or several
	Leaf	Triangular-ovate	Broadly ovate-triangular	Triangular-ovate	Triangular-ovate
	Bract	Absent	Absent, or 1–2	Absent, rarely 1	Absent
Fruit morphology	Size (mm)	4 × 2.5 (3.5–6 × 1.5–2.0)	6 × 3.2 (4–7 × 2–3.5)	5 × 2 (3–5.5 × 1–2.5)	6.08 × 2.06 (4.5–7.5 × 1.5–3.5)
	Shape	Ellipsoid	Elongated oval	Ellipsoid	Oblong
	Calyx teeth	Narrow triangular	Subulate	Narrow triangular	Subulate
	Hairy	Smooth, glabrous	Smooth, glabrous	Smooth, glabrous	Smooth, glabrous
Fruit transection	Shape	Pentagonal	Pentagonal	Subpentagonal	Semicircular
	Compressed degree	Laterally compressed	Laterally compressed	Laterally compressed	Laterally compressed
	Endosperm surface	Concave	Concave	Concave	Concave
Vitta number	Furrow	1–2	1–2	1–2	2–3
	Commissure	2–4	2–4	2–4	4–6
Pollen	Equatorial view	Rombiformis	Rombiformis	Rombiformis	Rombiformis
	Polar view	Subtriangular	Subtriangular	Subtriangular	Subtriangular
	Exine ornamentation	Short rod-like pseudo-cerebroid	Short rod-like pseudo-cerebroid	Short rod-like pseudo-cerebroid	Short rod-like pseudo-cerebroid
	Aperture	Goniotreme	Goniotreme	Goniotreme	Goniotreme
	P (μm)	26.53 (22.52–34.94)	34.63 (26.35–34.96)	30.89 (24.47–31.94)	24.52 (22.39–25.79)
	E (μm)	23.69 (16.25–24.10)	25.65 (17.78–26.20)	24.91 (16.78–25.10)	18.84 (15.01–20.60)
	P/E	1.12 (1.10–1.20)	1.35 (1.15–1.38)	1.24 (1.11–1.30)	1.30 (1.12–1.39)

### 3.2 Plastome features, plastome comparison, and hotspots identification

The size of four plastomes ranged from 155,169 bp (*C. peucedanifolia*) to 156,014 bp (*C. waltonii*). All plastomes exhibited a typical quadripartite structure, including one large single copy (LSC) region (85,079–85,461 bp) and one small single copy (SSC) region (17,794–17,915 bp) separated by two inverted repeats (IRs) regions (26,080–26,369 bp). The total Guanine and Cytosine (GC) content ranged from 37.7% to 37.8%, with the IR regions having the highest GC content (42.8%–43.0%) compared to the GC content in the LSC region (35.8%–35.9%) and SSC region (31.3%–31.5%) (**Supplementary Figure S2**; **Table 2**). All plastomes encoded 129 genes, comprising 84 protein-coding genes (PCGs), 37 transfer RNA (tRNA) genes, and eight ribosomal RNA (rRNA) genes (**Supplementary Table S5**).

**Table 2:** Features of the four plastomes.

Taxa	Total length (bp)				GC content (%)				Gene numbers			
	Size	LSC	SSC	IR	Total	LSC	SSC	IR	Total	Protein-coding genes	tRNA	rRNA
*Cyclorhiza waltonii*	156,014	85,411	17,915	26,344	37.7	35.9	31.5	42.8	129	84	37	8
*Cyclorhiza peucedanifolia*	155,169	85,160	17,794	26,107	37.8	35.9	31.5	43.0	129	84	37	8
*Cyclorhiza puana*	156,008	85,461	17,846	26,350	37.7	35.8	31.3	42.8	129	84	37	8
*Seseli purpureovaginatum*	155,651	85,079	17,856	26,357	37.7	35.9	31.4	42.8	129	84	37	8

In this study, the forward, palindromic, reverse, and complementary repeats were detected in these four species, and the total number of repeats was 214. Among these repeats, forward repeats were the most abundant (112), followed by palindromic repeats (98) and reverse repeats (4). Forward repeats and palindromic repeats were detected in all plastomes; reverse repeats were found in *C. waltonii*, *C. peucedanifolia*, and *S. purpureovaginatum*; and complementary repeats were not detected in any of the four plastomes. Moreover, the majority of these repeats were found in intergenic or intron regions (e.g., *rrn*5–*rrn*4.5, *rrn*4.5–*rrn*5, *pet*N–*psb*M, *ycf*2 intron, and *ycf*3 intron) (**Supplementary Figure S3A**; **Supplementary Table S6**). In addition, the total number of SSRs ranged from 48 (*C. waltonii*) to 59 (*S. purpureovaginatum*). The number of mono-repeats, di-repeats, tri-repeats, tetra-repeats, and hexa-repeats were 145, 39, 14, 20, and 1, respectively. All plastomes had the mono-repeats, di-repeats, tri-repeats, and tetra-repeats. The penta-repeats were not detected in all plastomes, and hexa-repeats only appeared in *S. purpureovaginatum*. SSRs were mostly distributed in the LSC region compared to the SSC and IR regions, and the majority of SSRs were distributed in the non-coding regions (**Supplementary Figure S3B**; **Supplementary Table S6**).

The IR boundaries of these four plastomes were highly consistent, and the adjacent genes were identical. The *rps*19 gene was located at the junction of LSC/IRb, with a length of 57 bp in the IRb region. The IRb/SSC junction region was located between the *ycf*1 gene and the *ndh*F gene. The length of the *ycf*1 gene located in the IRb region was 1,827–1,851 bp, and that of the *ndh*F gene was 17–67 bp away from the IRb/SSC borders. The *ycf*1 gene crossed the SSC/IRa boundary and had a length of 1,835–1,885 bp in the IRa region. The IRa/LSC boundary was located between the *rpl*2 gene and *trn*H gene, and the *rpl*2 gene and *trn*H gene were 115 bp and 3 bp away from the IRa/LSC borders, respectively (**Supplementary Figure S4**). Using the mVISTA program, we discovered that these four plastomes were highly conserved. Specifically, the protein-coding regions were more conserved than the non-coding regions, and the IR regions were more conserved than the LSC and SSC regions (**Supplementary Figure S5**). Furthermore, the Mauve result also revealed that these four plastomes were highly conserved and that no gene rearrangement or loss was found (**Supplementary Figure S6**).

To characterize the codon usage patterns across the four plastomes, we extracted and concatenated 53 protein-coding genes from each species. These protein sequences encoded 21,214–21,228 codons, with *C. peucedanifolia* being the most and *C. waltonii* being the least. Among them, Leu was the most abundant (2,230–2,240) and encoded by six codons (UUA, UUG, CUU, CUC, CUA, and CUG), whereas Trp was the least abundant (380–382) in all plastomes. The RSCU values of all codons ranged from 0.34 to 1.98 across the four plastomes, with RSCU values of 30 codons in each species being greater than 1 (**Supplementary Figure S7**; **Supplementary Table S7**).

**Figure 6:**
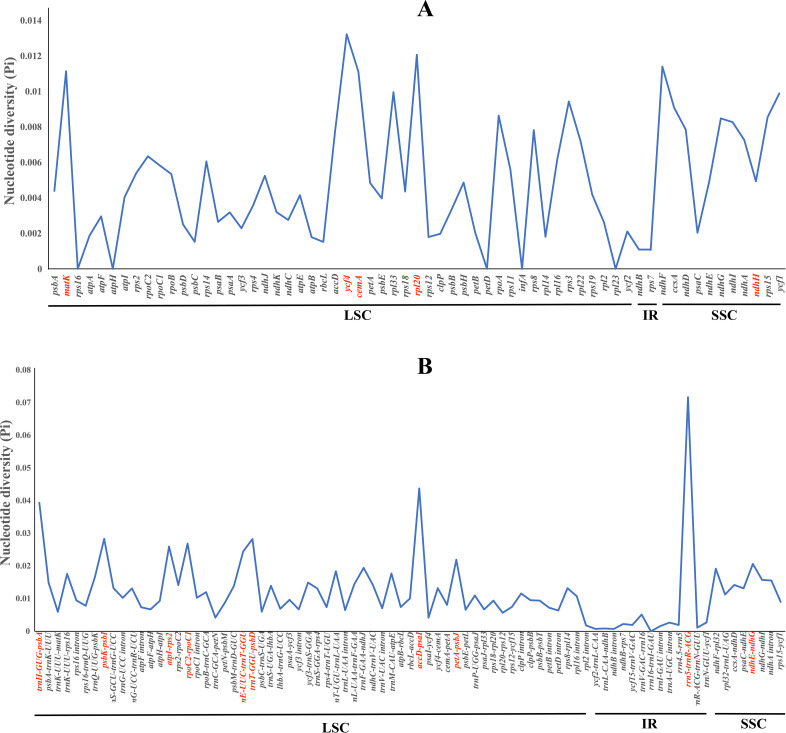
The nucleotide diversity (Pi) values among the four plastomes. **(A)** Coding regions. **(B)** Non-coding regions.

Furthermore, we computed the nucleotide diversity (Pi) of protein-coding genes, non-coding regions, and introns within the LSC, SSC, and IR regions (**Figure 6**; **Supplementary Table S8**). We identified five protein-coding genes (*cem*A, *mat*K, *ndh*F, *rpl*20, and *ycf*4) with relatively high nucleotide diversity (Pi > 0.01) (**Figure 6A**). Ten non-coding regions (*ndh*E–*ndh*G, *pet*A–*psb*J, *trn*E–*trn*T, *atp*I–*rps*2, *rpo*C2–*rpo*C1, *trn*T–*psb*D, *psb*K–*psb*I, *trn*H–*psb*A, *acc*D–*psa*I, and *rrn*5–*trn*R) also possessed high nucleotide diversity (Pi > 0.02) (**Figure 6B**).

### 3.3 Genetic diversity and structure

**Figure 7:**
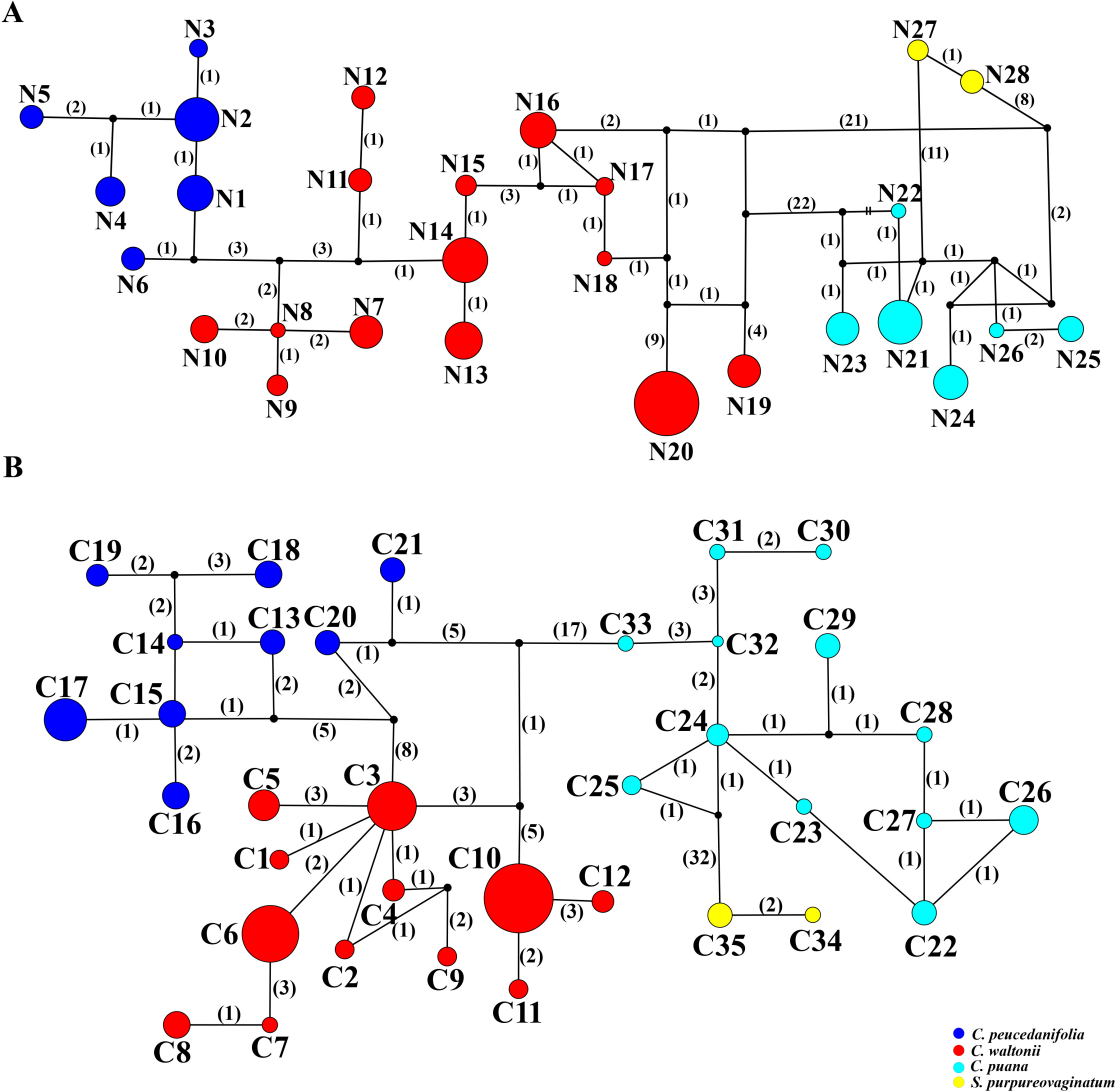
TCS networks for 28 ITS haplotypes and 35 cpDNA haplotypes. Each color represents a species. The size of circles in the network corresponds to the frequency of each haplotype. Numbers on the branches indicate the number of mutations between haplotypes. Small solid black circles denote hypothetic unsampled or extinct ancestral haplotypes. **(A)** ITS haplotypes. **(B)** cpDNA haplotypes.

In this study, ITS sequence and three cpDNA fragments (*mat*K, *trn*Q–*rp*s16, and *trn*D–*trn*T) were used to analyze 244 individuals from 27 populations. First, the haplotype distribution and genetic diversity were analyzed based on ITS data. The results showed that the aligned sequences of ITS were 600 bp, and GC content ranged from 53.1% to 55.3% (average 54.5%). A total of 66 polymorphic sites and 28 nuclear gene haplotypes (N1–N28) were detected, including six in *C. peucedanifolia* (N1–N6), fourteen in *C. waltonii* (N7–N20), six in *C. puana* (N21–N26), and two in *S. purpureovaginatum* (N27–N28), and no shared haplotype existed among different species. All species formed a monophyletic group in the ITS haplotype network (**Figure 7A**; **Supplementary Table S9**). The haplotype diversity (*H*d) was 0.941, and nucleotide diversity (π) was 0.03051 of ITS in the genus. As for these four species, the haplotype diversity (*H*d) and nucleotide diversity (π) were respectively 0.867 and 0.02090 in *C. waltonii*, 0.788 and 0.00354 in *C. peucedanifolia*, 0.770 and 0.00525 in *C. puana*, 0.556 and 0.00093 in *S. purpureovaginatum*. The haplotype diversity (*H*d) ranged from 0.000 to 0.002, and nucleotide diversity (π) ranged from 0.000 to 0.0103 of the population (**Supplementary Table S9**). Additionally, ITS data correlation analysis showed that the total gene diversity (*H*_T_) value (0.963) was higher than the average gene diversity within populations (*H*_S_) (0.335) at the genus level. The number of substitution types (*N*_ST_, 0.935) was higher than inter-population differentiation (*G*_ST_, 0.653). The gene flow (Nm) detected among all 27 populations was 0.06, and *C. peucedanifolia* possessed the highest Nm value (0.06) compared to *C. waltonii* (0.05) and *C. puana* (0.02) (**Table 3**). ITS AMOVA detected 62.51% of genetic variation occurring between species, 32.53% occurring between populations, and 4.97% occurring within populations. In addition, the *Cyclorhiza* species exhibited higher genetic variation between populations than within populations (**Table 4**).

**Table 3:** Genetic diversity and genetic differentiation based on ITS and cpDNA datasets.

Species	*H*_S_	*H*_T_	*G*_ST_	*N*_ST_	Nm
ITS					
*Cyclorhiza waltonii*	0.391 (0.0957)	0.897 (0.0444)	0.565 (0.0969)	0.822 (0.0659)	0.05
*Cyclorhiza peucedanifolia*	0.298 (0.1355)	0.916 (0.0353)	0.674 (0.1668)	0.841 (0.1158)	0.06
*Cyclorhiza puana*	0.166 (0.0795)	0.918 (0.0798)	0.819 (0.0924)	0.935 (0.0355)	0.02
*Seseli purpureovaginatum*	–	–	–	–	–
Total	0.335 (0.0637)	0.963 (0.0156)	0.653 (0.0652)	0.935 (0.0225)	0.06
cpDNA					
*C. waltonii*	0.432 (0.0724)	0.842 (0.0568)	0.487 (0.0609)	0.784 (0.0414)	0.10
*C. peucedanifolia*	0.404 (0.1169)	0.953 (0.0482)	0.576 (0.1232)	0.837 (0.0711)	0.06
*C. puana*	0.718 (0.0803)	0.970 (0.0255)	0.260 (0.0868)	0.466 (0.1003)	0.39
*S. purpureovaginatum*	–	–	–	–	–
Total	0.480 (0.0533)	0.950 (0.0234)	0.494 (0.0501)	0.917 (0.0136)	0.05

**Table 4:** Analysis of molecular variance (AMOVA) based on ITS and cpDNA data.

Species	Source of variation	*d.f.*	*SS*	*VC*	*PV* (%)	Fixation indices
ITS						
All species	Among species	3	1,248.304	7.68394	62.51	F_SC_: 0.86756
	Among populations within species	23	827.919	3.99851	32.53	F_ST_: 0.95035
	Within population	214	130.623	0.61039	4.97	F_CT_: 0.62507
*Cyclorhiza waltonii*	Among populations	14	719.790	5.75843	86.21	F_ST_: 0.86207
	Within population	117	107.801	0.92137	13.79	
*Cyclorhiza peucedanifolia*	Among populations	5	37.337	0.85356	70.85	F_ST_: 0.70854
	Within population	45	15.800	0.35111	29.15	
*Cyclorhiza puana*	Among populations	4	70.792	1.79928	94.28	F_ST_: 0.94284
	Within population	44	4.800	0.10909	5.72	
*Seseli purpureovaginatum*	Among populations	–	–	–	–	–
	Within population	–	–	–	–	
All samples	Among populations	26	2,076.223	8.89547	93.58	F_ST_: 0.93579
	Within population	214	130.623	0.61039	6.42	
cpDNA						
All species	Among species	3	1,329.932	9.48284	69.38	F_SC_: 0.76839
	Among populations within species	23	618.734	3.21599	23.53	F_ST_: 0.92908
	Within population	192	186.115	0.96935	7.09	F_CT_: 0.69379
*C. waltonii*	Among populations	14	405.754	3.49765	83.12	F_ST_: 0.83116
	Within population	107	76.024	0.71051	16.88	
*C. peucedanifolia*	Among populations	5	183.435	4.09735	84.50	F_ST_: 0.84497
	Within population	48	36.083	0.75174	15.50	
*C. puana*	Among populations	3	29.544	0.71591	76.2223.78	F_ST_: 0.23776
	Within population	41	71.150	2.29516	23.78	
*S. purpureovaginatum*	Among populations	–	–	–	–	–
	Within population	–	–	–	–	
All samples	Among populations	26	1,948.666	9.15457	90.43	F_ST_: 0.90425
	Within population	192	186.115	0.96935	9.57	

*d.f.*, degree of freedom; *SS*, Square Sum; *VC*, Variance Component; *PV*, Probability Variation.

Moreover, the haplotype distribution and genetic diversity were analyzed based on three cpDNA fragment data. The total length of the aligned sequences was 2,116 bp (*mat*K: 745 bp, *trn*Q-*rps*16: 662 bp, *trn*D-*trn*T: 709 bp). A total of 90 polymorphic sites and 35 chloroplast haplotypes (C1–C35) were detected, including twelve in *C. waltonii* (C1–C12), nine in *C. peucedanifolia* (C13–C21), twelve in *C. puana* (C22–C33), and two in *S. purpureovaginatum* (C34–C35), and no shared haplotype existed among different species (**Figure 7B**; **Supplementary Table S9**). Simultaneously, all species form a monophyletic group in the haplotype network (**Figure 7B**). At the genus *Cyclorhiza* level, the haplotype diversity (*H*d) was 0.9324, and nucleotide diversity (π) was 0.00927. At the species level, the haplotype diversity (*H*d) and nucleotide diversity (π) were respectively 0.815 and 0.00379 in *C. waltonii*, 0.869 and 0.00391 in *C. peucedanifolia*, 0.916 and 0.00289 in *C. puana*, and 0.476 and 0.00045 in *S. purpureovaginatum*. The haplotype diversity (*H*d) ranged from 0.000 to 0.867, and nucleotide diversity (π) ranged from 0.000 to 0.00354 of the population (**Supplementary Table S9**). At the genus *Cyclorhiza* level, the total gene diversity (*H*_T_) value (0.950) was higher than the average gene diversity within populations (*H*_S_) (0.480). In addition, the number of substitution types (*N*_ST_, 0.917) was higher than inter-population differentiation (*G*_ST_, 0.494). The gene flow (Nm) detected among all 27 populations was 0.05, and *C. puana* possessed the highest Nm value (0.39) compared to *C. waltonii* (0.10) and *C. peucedanifolia* (0.06) (**Table 3**). cpDNA AMOVA detected 69.38% of genetic variation occurring between species, 23.53% occurring between populations, and 7.09% occurring within populations. Furthermore, the *Cyclorhiz* species exhibited higher genetic variation between populations than within populations (**Table 4**).

### 3.4 Phylogeny reconstruction and divergence time estimation

**Figure 8:**
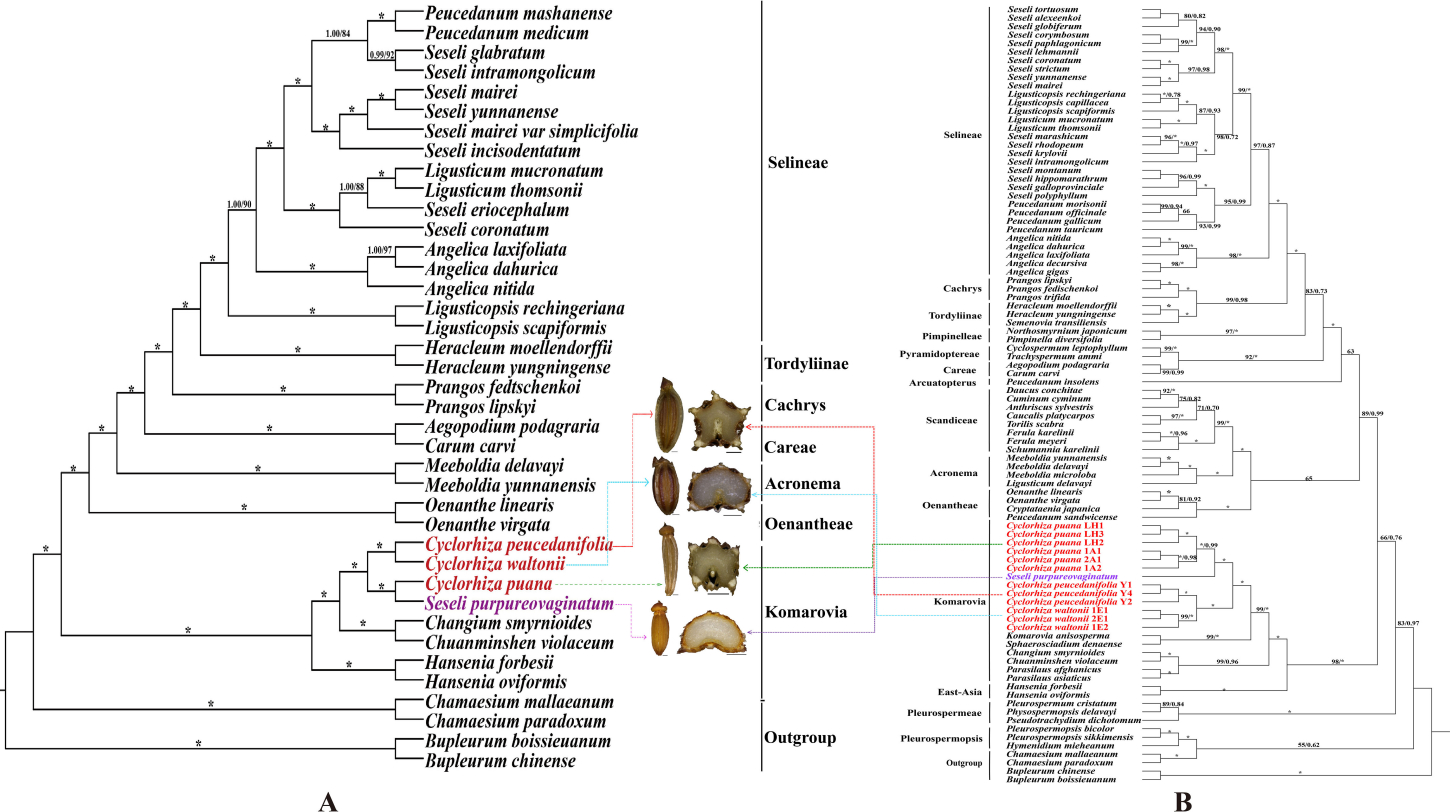
Phylogeny from maximum likelihood (ML) and Bayesian inference (BI) analyses. The bootstrap values (BS) of ML and posterior probabilities (PP) of BI are listed at each node (* represents the node = 100/1.00; - represents the node < 50/0.50). **(A)** CDS tree. **(B)** ITS tree.

**Figure 9:**
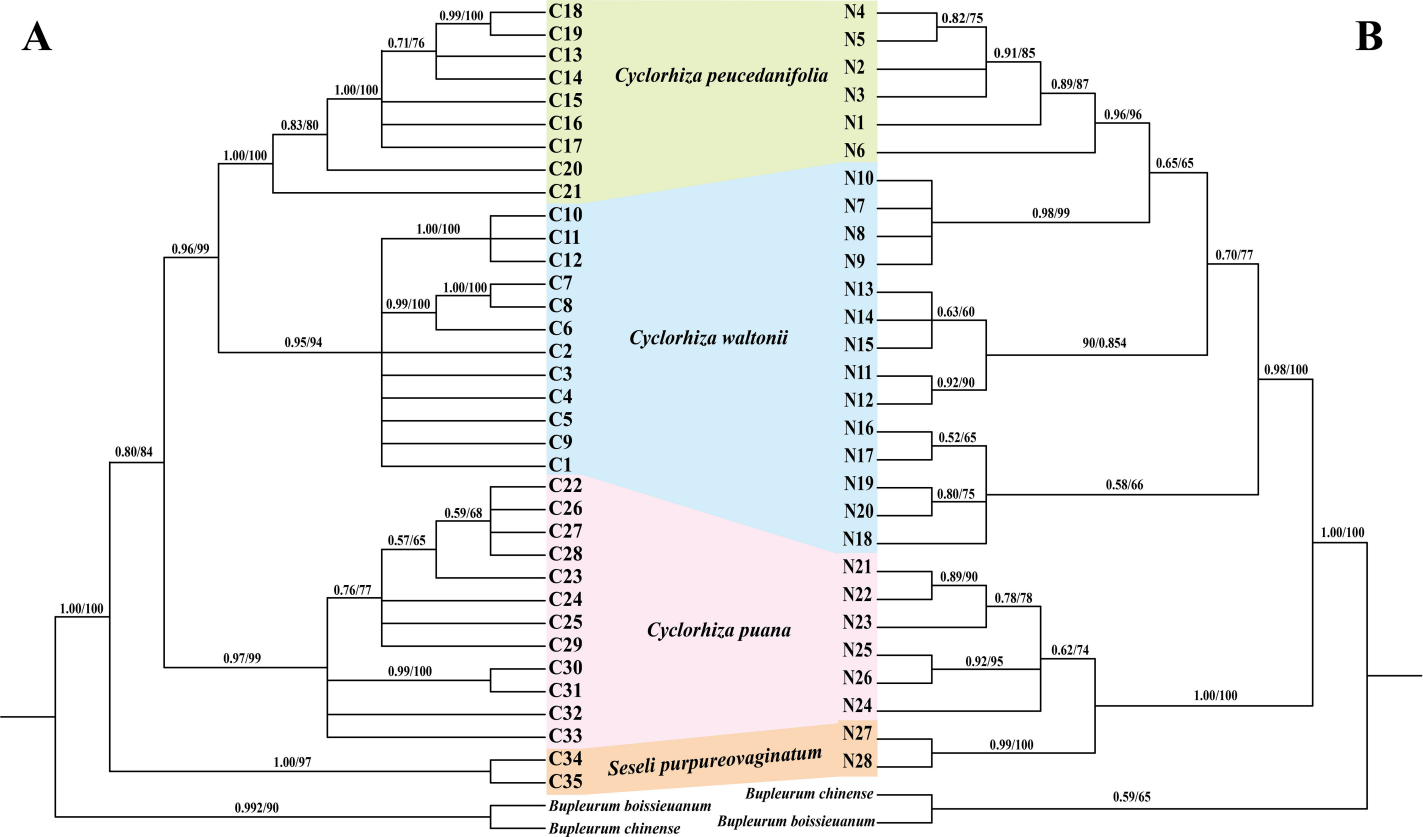
Phylogeny from maximum likelihood (ML) and Bayesian inference (BI) analyses. The bootstrap values (BS) of ML and posterior probabilities (PP) of BI are listed at each node. Haplotypes from different species are marked with different colors. **(A)** cpDNA haplotypes. **(B)** ITS haplotypes.

We used three molecular datasets: 39 complete plastomes, 92 ITS sequences, and haplotypes (28 ITS haplotypes and 35 cpDNA haplotypes) to reconstruct the phylogenetic trees (**Figures 8**, **9**). All topologies were highly consistent, which strongly supported that the *Cyclorhiza* species clustered together, belonging to the Komarovia clade. *S. purpureovaginatum* was nested within the genus *Cyclorhiza* and also located in the Komarovia clade, while other *Seseli* members clustered in the Selineae clade and were distant from *S. purpureovaginatum*. In addition, the phylogenetic results also robustly indicated that *C. waltonii* was sister to *C. peucedanifolia* (CDS trees: PP = 1.00, BS = 100; ITS tree: PP = 1.00, BS = 100), and *C. puana* clustered with *S. purpureovaginatum* (CDS trees: PP = 1.00, BS = 100; ITS tree: PP = 1.00, BS = 100) (**Figure 8**). Furthermore, the haplotype phylogenetic trees showed that *C. waltonii* populations were sisters to the *C. peucedanifolia* populations (ITS haplotypes: PP = 0.96, BS = 99; cpDNA haplotypes: PP = 0.65, BS = 65), and *C. puana* populations were sisters to *S. purpureovaginatum* populations (ITS haplotypes: PP = 1.00, BS = 100; cpDNA haplotypes: PP = 1.00, BS = 100) (**Figure 9**), and the haplotype phylogenetic trees were consistent with the haplotype network (**Figures 7**, **9**).

**Figure 10:**
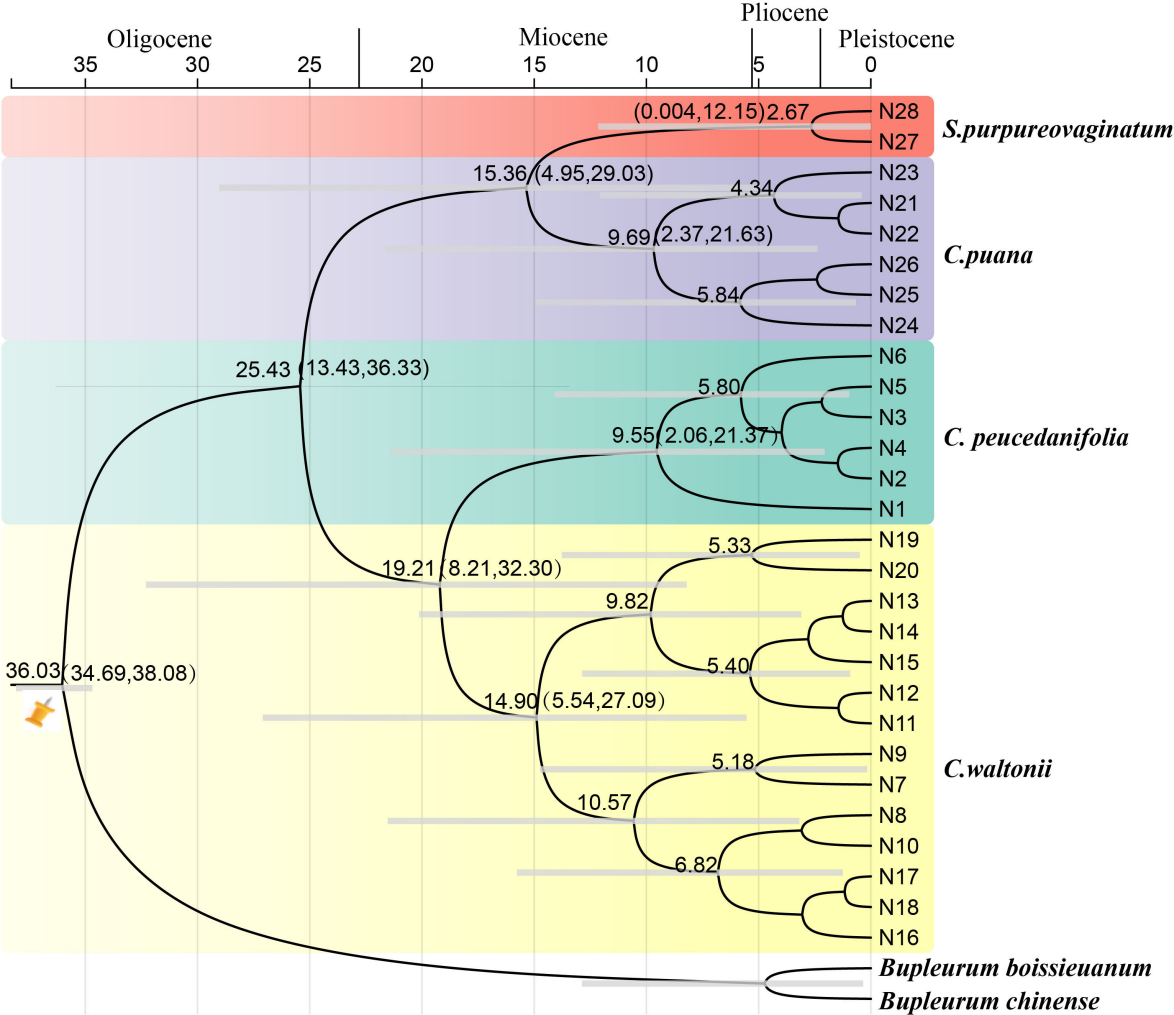
The divergence time estimation based on ITS sequence. The maximum credibility tree from the divergence times estimated with BEAST. The 95% highest posterior density (HPD) estimates for each well-supported clade are represented by bars. Orange nails indicate the calibration points for the molecular dating.

The molecular dating analyses showed that the genus *Cyclorhiza* originated in the late Eocene [36.03 Ma; 95% highest posterior density (HPD): 34.69–38.08 Ma] and the diversification of the genus occurred at late Oligocene, with the age 25.43 Ma (95% HPD: 13.43–36.33 Ma) (**Figure 10**). In detail, the divergence time between *C. waltonii* and *C. peucedanifolia* was estimated to be 19.21 Ma (95% HPD: 8.21–32.30 Ma) at the early Miocene period, and the divergence time between *C. puana* and *S. purpureovaginatum* was estimated to be 15.36 Ma (95% HPD: 4.95–29.03 Ma) at the middle Miocene period. Moreover, the populations of these species continued to differentiate between the Miocene and Pleistocene periods (**Figure 10**).

## 4 Discussion

### 4.1 The micromorphological taxonomic value of fruits and pollens

Cremocarp is a unique fruit in the Apiaceae family, which is widely used in taxonomic studies of many genera of Apiaceae, such as in *Sanicula* L. (Song et al., 2024a, c), *Ferula* L. (Qin et al., 2023), *Sinocarum* Wolff ex Shan & Pu (Xiao et al., 2021), *Seseli* L. (Cai et al., 2022), and *Acronema* Edgew. (Chen et al., 2024). In this study, the fruit micromorphology and anatomical features of the *Cyclorhiza* species were highly similar. However, some differences (fruit size and calyx teeth) were detected, such as *C. peucedanifolia* having the largest fruit size, while *C. puana* had the smallest fruit size, and the calyx teeth of *C. peucedanifolia* were subulate, whereas those of *C. waltonii* and *C. puana* were narrow triangular. These significant differences allow them to be easily distinguishable from each other. Moreover, we also found that the fruit of *S. purpureovaginatum* shared some similarities with *Cyclorhiza* species, such as a long-ellipsoid shape, smooth and glabrous, slightly laterally compressed, with the endosperm commissural face concave, ribs prominent, ribs equal, and shortly keeled, which was consistent with the *C. puana*; its subulate calyx teeth was accordant with *C. peucedanifolia*, and its vitta numbers overlapped with those of the *Cyclorhiza* species (**Table 1**). All these findings implied that *S. purpureovaginatum* belonged to the genus *Cyclorhiza* and should be transferred from the genus *Seseli* to *Cyclorhiza*. Therefore, the fruit’s micromorphological and anatomical features implied that the genus *Cyclorhiza* was a monophyletic group after including *S. purpureovaginatum*.

The morphology and micromorphology of pollen are mainly controlled by genes and are rarely influenced by the environment, thus possessing strong conservation and stability (Peng et al., 2018; Zhou et al., 2022). The pollen morphology (pollen size, outer wall ornamentation, and aperture characteristics) is of great significance in revealing the origin, evolution, and phylogenetic relationships of plants (Palynological Section, 1960; Wang and Wang, 1983; Zhang and Wang, 2007). In taxonomic studies of the Apiaceae family, the rich and diverse pollen characteristics can also provide a valuable reference for exploring the phylogenetic relationships and evolutionary status between species (Qin and Shen, 1990; Meng et al., 2004; Chen et al., 2007; Wang et al., 2012; Zhang et al., 2018; Jardine et al., 2022). In this study, we found that the pollen of *Cyclorhiza* species exhibited highly similar characteristics, such as rombiformis equatorial view, subtriangle polar view, short rod-like pseudo-cerebroid of exine ornamentation of the equatorial view, and angular germinal aperture, which were accordant with the study of Shu and Sheh (2001). Nevertheless, the pollen size of three *Cyclorhiza* species displayed differences. For example, *C. peucedanifolia* had the largest size, while *C. waltonii* had the smallest size, so they can be clearly distinguished from each other by pollen size. Meanwhile, the rombiformis pollen shape and angular germinal aperture indicated that *Cyclorhiza* was a relatively primitive group in the Apiaceae family (Zhang et al., 2013). Furthermore, we found that the pollen of *S. purpureovaginatum* were highly similar to those of *Cyclorhiza* species, implying that *S. purpureovaginatum* was indeed a member of the *Cyclorhiza* and should be transferred to the genus. Thus, pollen morphology once again indicated that the genus *Cyclorhiza* was a natural group after including *S. purpureovaginatum*.

### 4.2 Plastome evolution

All plastomes displayed a typical quadripartite structure that may be related to the stability of plastome function (Liu et al., 2022). No gene rearrangement or loss was detected in these four plastomes, which was consistent with other genera of Apiaceae (Wen et al., 2021; Liu et al., 2022; Ren et al., 2022; Gui et al., 2023; Qin et al., 2023; Song et al., 2023; Chen et al., 2024; Guo et al., 2024; Song et al., 2024b, c). Moreover, the genome size, GC content, IR boundaries, patterns of codon bias, and SSRs were very similar among these plastomes, which showed that these four plastomes were highly conserved. These findings also suggested that *S. purpureovaginatum* was indeed a member of the genus *Cyclorhiza* and implied the monophyly of the *Cyclorhiza* after including *S. purpureovaginatum*.

### 4.3 Genetic diversity and structural analyses

The genetic diversity of species plays an important role in their adaptability and survival ability, which is the result of multiple factors working together (Soltis, 1991). The higher the level of genetic diversity of a species, the more advantageous it is for species to adapt to complex and changing environments. Conversely, species with lower levels of genetic diversity have more difficulty adapting to environmental changes, which may even lead to species extinction (Avise and Hamrick, 1996; Petit et al., 2005). In this study, we found that both datasets showed high levels of overall genetic diversity (ITS: *H*_T_ = 0.963, cpDNA: *H*_T_ = 0.950) in the genus *Cyclorhiza*, and the population genetic differentiation coefficient was significantly higher than the geographic population differentiation coefficient, which indicated that the genus *Cyclorhiza* had a significant lineage geographic structure. Thus, we speculated that the reasons for the high level of genetic diversity in the genus *Cyclorhiza* may include the following. i) Long-term evolutionary history: as a basal group in the subfamily Apioideae of the family Apiaceae (Zhou et al., 2009; Wen et al., 2021), the genus *Cyclorhiza* had undergone many geological and climatic events over its long evolutionary history, leading to the accumulation of a large amount of genetic variation. ii) Complex terrain and habitat: *Cyclorhiza* plants are widely distributed in HHM region (Sheh and Shan, 1980; Zhou et al., 2021). These regions have complex terrains, including alpine meadows, alpine meadows, broad-leaved forests, rocky landforms, river valleys, and streams, as well as unique climate phenomena such as the Foehn effect (Zheng et al., 2021), which often occurs in these areas. The diversity of terrain and climate promotes the differentiation between populations and increases the likelihood of genetic drift, thereby accumulating rich genetic variation. iii) Sexual reproduction mode: the *Cyclorhiza* plants belong to sexual reproduction, which can maintain the genetic stability of the species, increase the possibility of genetic variation, and further promote the production of genetic diversity.

The diversity of haplotypes (ITS: 0.941, cpDNA: 0.9324) was relatively high at the level of the genus *Cyclorhiza* and three *Cyclorhiza* species (*C. waltonii*, ITS: 0.867, cpDNA: 0.815, *C. peucedanifolia*, ITS: 0.788, cpDNA: 0.869, and *C. puana*, ITS: 0.770, cpDNA: 0.916). However, *S. purpureovaginatum* had a low diversity of haplotypes (ITS: 0.556, cpDNA: 0.476) (**Table 4**), which may be caused by *S. purpureovaginatum* possessing only one population, and the data were not comprehensive enough. Therefore, it is necessary to increase the sample size of the *S. purpureovaginatum* population to avoid this problem in future research. TCS network showed that there were no shared haplotypes among these four species, and most haplotypes were limited to one or adjacent populations. We speculated that the species of this genus remained isolated during both glacial and interglacial periods due to a combination of species specificity and environmental climate factors. On the one hand, *Cyclorhiza* plants are distributed in the HHM region (Sheh and Shan, 1980; Pimenov, 2017; Zhou et al., 2021). Numerous high-altitude mountain ranges are separated by river canyons, and the rise of monsoons on the mountains has caused significant climate change, including the Foehn effect, which severely hindered gene exchange between species of the genus *Cyclorhiza*. On the other hand, the limited pollen/seed dispersal ability of the *Cyclorhiza* species can also affect this process. Similar results were also found in the genus *Chamaesium* H. Wolff (Zheng et al., 2021).

The AMOVA results based on ITS data and cpDNA fragments indicated that the majority of genetic variation occurred between species (ITS 62.51%, cpDNA: 69.38%), while genetic variation within populations (ITS: 4.97%, cpDNA: 7.09%) was rare. The phenomenon was subjected to geographic barriers and species specificity in the HHM region, which increased the genetic variation between different species (Liu et al., 2006). Meanwhile, this region possessed complex and diverse habitats that increased the selection pressure, and fragmented habitats may limit gene flow between populations (Zheng et al., 2021). In addition, the limited ability of pollen/seed dispersal in the genus *Cyclorhiza* restricted the gene exchange between populations and increased their genetic differentiation level.

### 4.4 Phylogenetic analyses

The phylogenetic analyses based on plastome data, ITS sequences, and haplotypes indicated that *Cyclorhiza* species clustered into a separate clade, belonging to the Komarovia clade, which was consistent with the previous studies (Zhou et al., 2008). Interestingly, *S. purpureovaginatum* nested within *Cyclorhiza*, belonging to the Komarovia clade, and other members of *Seseli* were located in the Selineae clade, which was distant from the Komarovia clade (**Figures 8**, **9**). The previous molecular studies (ITS, *rps*16 intron, *rpl*16 intron, and plastome) had proved that *Seseli* was not a monophyletic group, and its taxonomy has faced extreme challenges (Downie et al., 2000; Spalik et al., 2004; Zhou et al., 2009; Downie et al., 2010; Cai et al., 2022). To further investigate the phylogeny and taxonomy of *Seseli*, our team established “a narrow sense” of *Seseli* based on molecular, including the type species of *Seseli* (*S. tortuosum*), which was located in the Selineae clade. The morphological data also supported “a narrow sense” of *Seseli*, such as leaf segments linear to lanceolate, bracts nearly absent, bracteoles linear to lanceolate, rays unequal, calyx teeth very minute, mericarps ovoid or oblong, and ribs prominent (Cai et al., 2022). In this study, our phylogenetic analyses showed *S. purpureovaginatum* was distant from the generitype species *S. tortuosum*, and it does not belong to “a narrow sense” of *Seseli*. It also had different features from *S. tortuosum*, such as being herbaceous and glabrous, with erect stems branching above, no bracts and bracteoles, and yellow petals. Therefore, the taxonomic positions of *S. purpureovaginatum* need to be re-evaluated. Therefore, the phylogenetic results strongly suggested that *S. purpureovaginatum* should be transferred from the genus *Seseli* to *Cyclorhiza*. The morphological data and plastome analyses also confirmed the rationality of phylogenetic analyses (**Supplementary Figures S2**–**S6**; **Tables 1**, **2**). The molecular evidence, morphological data, and plastome analyses justified the monophyly of the genus *Cyclorhiza* after including *S. purpureovaginatum*. Furthermore, we also clarified the relationship among these four species: *C. waltonii* was sister to *C. peucedanifolia*, and *C. puana* clustered with *S. purpureovaginatum*. The haplotype phylogenetic trees (ITS and cpDNA) justified that the populations of each species gathered together without crossing each other. It also justified that the inter-population differentiation may be due to limited gene flow, which was accordant with the previous studies (Zhang et al., 2013; Meng et al., 2017; Xie et al., 2018).

Finally, we clarified the generic limits of *Cyclorhiza* based on the robust phylogenetic framework, morphological characteristics, and plastome comparative analyses, that is, herbaceous and glabrous, carrot-like roots with prominent annular scars when old, stem fistulose, erect, branched above, base clothed in purplish-brown remnant sheaths, absence of bracts and bracteoles, yellow petals, fruits ovoid or ellipsoid, smooth, slightly laterally compressed, mericarps subpentagonal in cross-section, and seed face deeply sulcate or concave. The abovementioned morphological characteristics are unique to *Cyclorhiza*, which can be easily distinguished from other genera of Apiaceae. Hence, we provided a species classification key index for the genus *Cyclorhiza*.

### 4.5 Origin and diversification of *Cyclorhiza*

The HHM region is a natural laboratory for investigating the processes of ecological speciation (Yu et al., 2023). Due to the fact that the genus *Cyclorhiza* is endemic to the HHM region (Pimenov, 2017; Zhou et al., 2021), it is of great value to study its origin and differentiation. Our analysis based on date estimation revealed that the genus occurred in the late Eocene (36.03 Ma, 95% HPD: 34.69–38.08 Ma). This period was associated with the early uplift of the southern QTP (Barba-Montoya et al., 2018), which led to the development of alpine dwelling habitats and the independent evolution of plant lineages, such as Chamaesieae, Bupleureae, and Pleurospermeae (Wen et al., 2021). A previous study demonstrated that the initial uplift of the QTP occurred in the middle Eocene to late Eocene (45–35 Ma) (Barba-Montoya et al., 2018), and the monolithic uplift of the QTP led to the uplift of HDM. The uplift events have caused drastic habitat fragmentation and heterogeneity, which played important roles in promoting the formation and differentiation of species, such as in *Sanicula* L. (Song et al., 2024b) and *Saxifraga* Tourn. ex L. (Ebersbach et al., 2017). Therefore, we hypothesized that the origin of the genus *Cyclorhiza* was closely related to the early uplift of the QTP and HDM. In addition, the diversification of *Cyclorhiza* occurred at the late Oligocene, with the age 25.43 Ma (95% HPD: 13.43–36.33 Ma). During the Oligocene (33.9–23.03 Ma), the climate seemed to be temperate, and many regions were nearly tropical. Grasslands expanded and forested regions dwindled (Britannica, 2020). As the uplift continued, the QTP and its adjacent mountain acted as orographic barriers to the Asian atmospheric circulation, which directly led to the formation of the monsoon climate (Zheng et al., 2021). Therefore, we speculated that the differentiation of the genus *Cyclorhiza* was largely influenced by the colonization of the newly available climate and terrain.

### 4.6 Taxonomic treatment

*Cyclorhiza purpureovaginata* (R. H. Shan & M. L. Sheh) J.Cai, X.Y.M.Aou & S.D.Zhou, comb. nov.

≡ *Seseli purpureovaginatum* R. H. Shan & M. L. Sheh in Acta Phytotax. Sin. 18 (3): 377.

Type: China. Xizang: Biru County, Baiga Valley, in sunny mountain slopes and crevices, ca. 3800 m, 9 September 1976, Qinghai-Xizang Exped. 11346 (holotype PE! barcode PE00935541) (**Supplementary Figure S1**) (Wolff, 1925; Sheh and Shan, 1980).

Description: Perennial herbs, 25–50 cm tall, monocarpic, glabrous throughout. The stem is solitary, mostly upright, sturdy, and sparsely branched near the top. Basal leaves are numerous and characterized by dark purple scarious margins. The leaf blade is ovate, measuring 6–10 cm in length and 3–5 cm in width, divided into two pinnate segments. The pinnae are shortly petiolulate, and the ultimate segments are linear or linear-elliptic, 4–10 mm in length and 1.5–5 mm in width. The ultimate segments resemble the basal leaves. The synflorescence is dichotomously branched, with a few loose compound umbels that are 2.5–5 cm in diameter. No bracts or bracteoles are present, and three to five rays measure 1.5–3 cm. The calyx teeth are very small or absent. The petals are smooth and white. The stylopodium is cone-shaped, and the styles are short. The fruit is pale yellow and oblong, with a rounded-pentagonal cross-section, measuring 3.5–5 mm in length and 2–3 mm in width. It is glabrous with prominent ribs of equal size and a slight keel. Each furrow contains 2–3 vittae, while there are 4 vittae on the commissure.

Distribution and habitat in China: *S. purpureovaginatum* is endemic to southwestern China (east Xizang). It grows in alpine meadows on sunny mountain slopes at an elevation of 3,800–4,000 m.

Additional specimens examined: China-Xizang: Biru Xian, alt. 3,800 m, 9 September 1976, Qinghai-Xizang Exped. 11346 (holotype PE); Baiga Village, 31°20′N, 94°04′E, 3,986 m, 31 July 2022, J.Cai & J.Q.Lei CJ202208040101 (SZ).

Key to species of *Cyclorhiza*

1a. The leaf sheath is a deep, dark purple, with slightly keeled ribs, containing 2–3 vittae in each furrow and 4–6 on the commissure.………………………………*C. purpureovaginata*
  1b. The leaf sheath is a light shade of dark purple, with prominent, almost narrowly winged filiform ribs. There are 1–2 vittae in each furrow and 2–3 on the commissure………2
  2a. The final leaf segments are shaped from ovate-oblong to linear-lanceolate, measuring 20 to 60 mm in length and 3 to 10 mm in width.…………………………………*C. peucedanifolia*
  2b. Ultimate leaf segments linear, 2–20 × 0.5–6 mm…………3
  3a. Ultimate leaf segments 4–20 × 2–6 mm; rays unequal; stylopodium low-conic; seed face deeply sulcate........*C. waltonii*
  3b. Ultimate leaf segments 2–4 × 0.5–1 mm; rays subequal; stylopodium ob-conic; seed face slightly concave.....…*C. puana*

## 5 Conclusion

In this study, we found that *Cyclorhiza* plants and *S. purpureovaginatum* shared similar morphological characteristics in root, stem, bracts, bracteoles, petals, fruits, and pollens. These evidence strongly supported the monophyly of the genus *Cyclorhiza* after including *S. purpureovaginatum*. In addition, we first sequenced and assembled the *Cyclorhiza* plastomes and performed comprehensive comparative analyses for the genus. The results revealed that the genome size, GC content, IR boundaries, patterns of codon bias, and SSRs were very similar among these plastomes, which showed that these four plastomes were highly conserved. These findings also suggested that *S. purpureovaginatum* was indeed a member of the genus *Cyclorhiza* and implied the monophyly of the *Cyclorhiza* after including *S. purpureovaginatum*. Nevertheless, we selected 15 mutation hotspots regions (*cem*A, *mat*K, *ndh*F, *rpl*20, *ycf*4, *ndh*E–*ndh*G, *pet*A–*psb*J, *trn*E–*trn*T, *atp*I–*rps*2, r*po*C2–*rpo*C1, *trn*T–*psb*D, *psb*K–*psb*I, *trn*H–*psb*A, *acc*D–*psa*I, and *rrn*5–*trn*R) as potentially strong DNA barcodes in *Cyclorhiza* species identification. The phylogenetic analyses of three datasets (plastome data, ITS sequences, and haplotypes) showed that *Cyclorhiza* species clustered into a separate clade, belonging to the Komarovia clade, and *S. purpureovaginatum* nested within *Cyclorhiza*. In detail, *C. waltonii* formed a clade with *C. peucedanifolia*, and *C. puana* clustered with *S. purpureovaginatum*. Thus, phylogenetic analyses also supported the monophyly of the genus *Cyclorhiza* after transferring *S. purpureovaginatum* into the genus. Then, we clarified the generic limits of *Cyclorhiza* based on morphological evidence, plastome comparative analyses, and phylogenetic analyses and provided a species classification key index for the genus. Furthermore, phylogeography analyses showed that the genus *Cyclorhiza* possessed high genetic diversity, and the population genetic differentiation coefficient was significantly higher than the geographic population differentiation coefficient, which indicated that the genus *Cyclorhiza* had a significant lineage geographic structure. Finally, the divergence time estimation showed that the genus *Cyclorhiza* originated in the late Eocene, which was closely related to the early uplift of the QTP and HDM. The diversification of the genus occurred in the late Oligocene, which was largely influenced by the colonization of the newly available climate and terrain. In conclusion, the study investigated the morphology, phylogeography, phylogeny, taxonomy, and evolution of the genus *Cyclorhiza* for the first time and provided valuable references for other genera of Apiaceae.

## Data Availability

The original contributions presented in the study are included in the article/**Supplementary Material**. Further inquiries can be directed to the corresponding author.

## Supplementary material

Supplementary Figure 1 The features of *Seseli purpureovaginatum.* **(A)** isolectotype (E00935541); **(B)** plant; **(C)** leaf; **(D)** fruit.

Supplementary Figure 2 Plastome maps of *Cyclorhiza* members and *S. purpureovaginatum*. Genes shown outside of the outer layer circle are transcribed clockwise, while those insides are transcribed counterclockwise. The genes belonging to diferent functional groups are color-coded. The dark gray area of the inner circle denotes the GC content of plastome.

Supplementary Figure 3 Analyses of repeats in the four plastomes. **(A)** Total number of four repeat types; **(B)** Total number of SSRs.

Supplementary Figure 4 Comparison of IR boundaries among four plastomes.

Supplementary Figure 5 mVISTA alignment for four plastomes with *C. waltonii* as the reference.

Supplementary Figure 6 Mauve alignment of four plastomes. Local collinear blocks within each alignment are represented by blocks of the same color connected with lines.

Supplementary Figure 7 The RSCU values of all concatenated protein-coding genes for four plastomes. Color key: higher RSCU values were denoted in red, while lower RSCU values were indicated in blue. * represented the terminator codons.
