# Supplementary figures and images for: Morphology, phylogeography, phylogeny, and taxonomy of Cyclorhiza (Apiaceae)
Source: Front Plant Sci. 2025 Jan 8;15:1504734. doi: 10.3389/fpls.2024.1504734 (PMC11750748; doi:10.3389/fpls.2024.1504734)

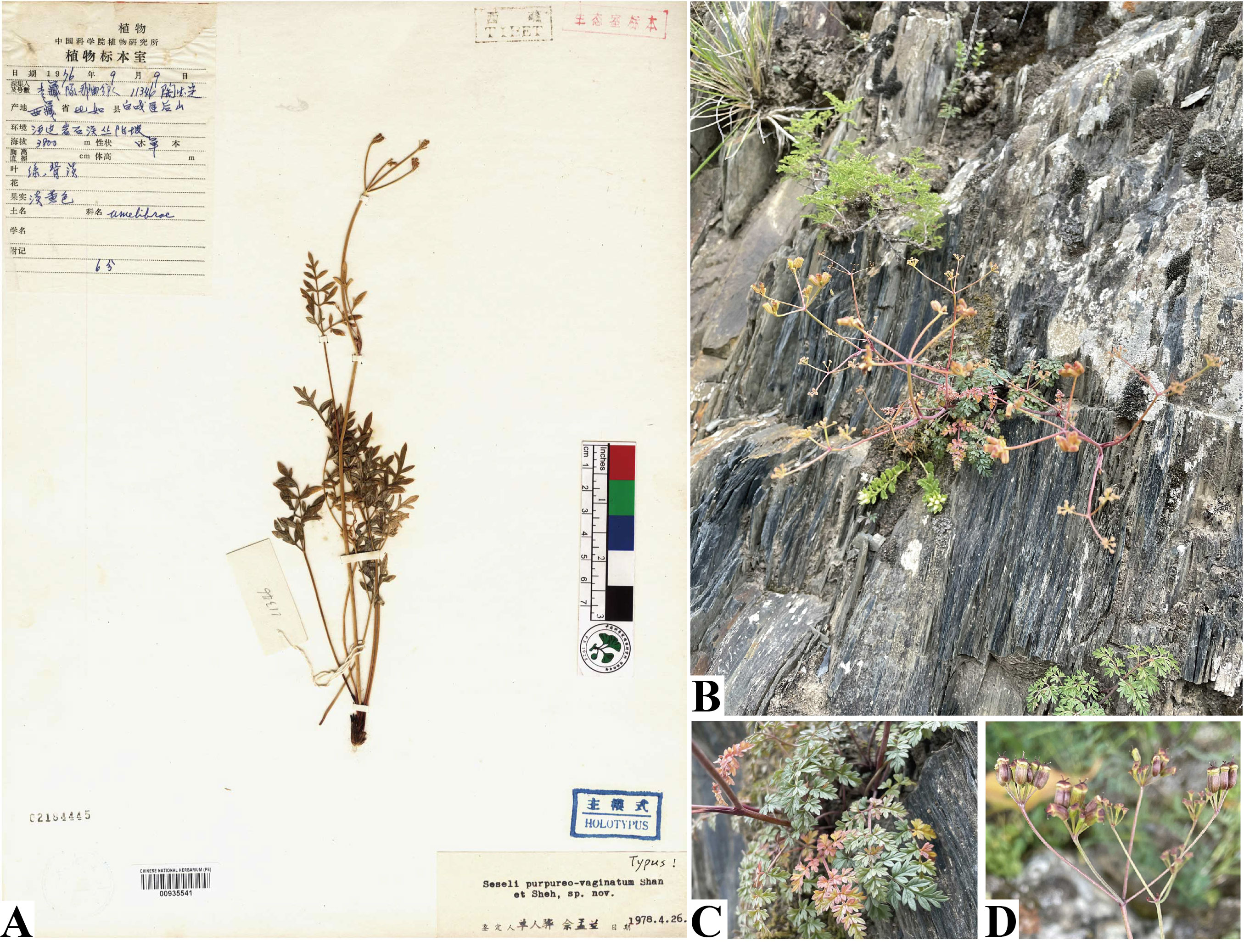

Supplement: Supplementary Figure 1 — The features of Seseli purpureovaginatum. (A) isolectotype (E00935541); (B) plant; (C) leaf; (D) fruit. [file Image1.jpeg]

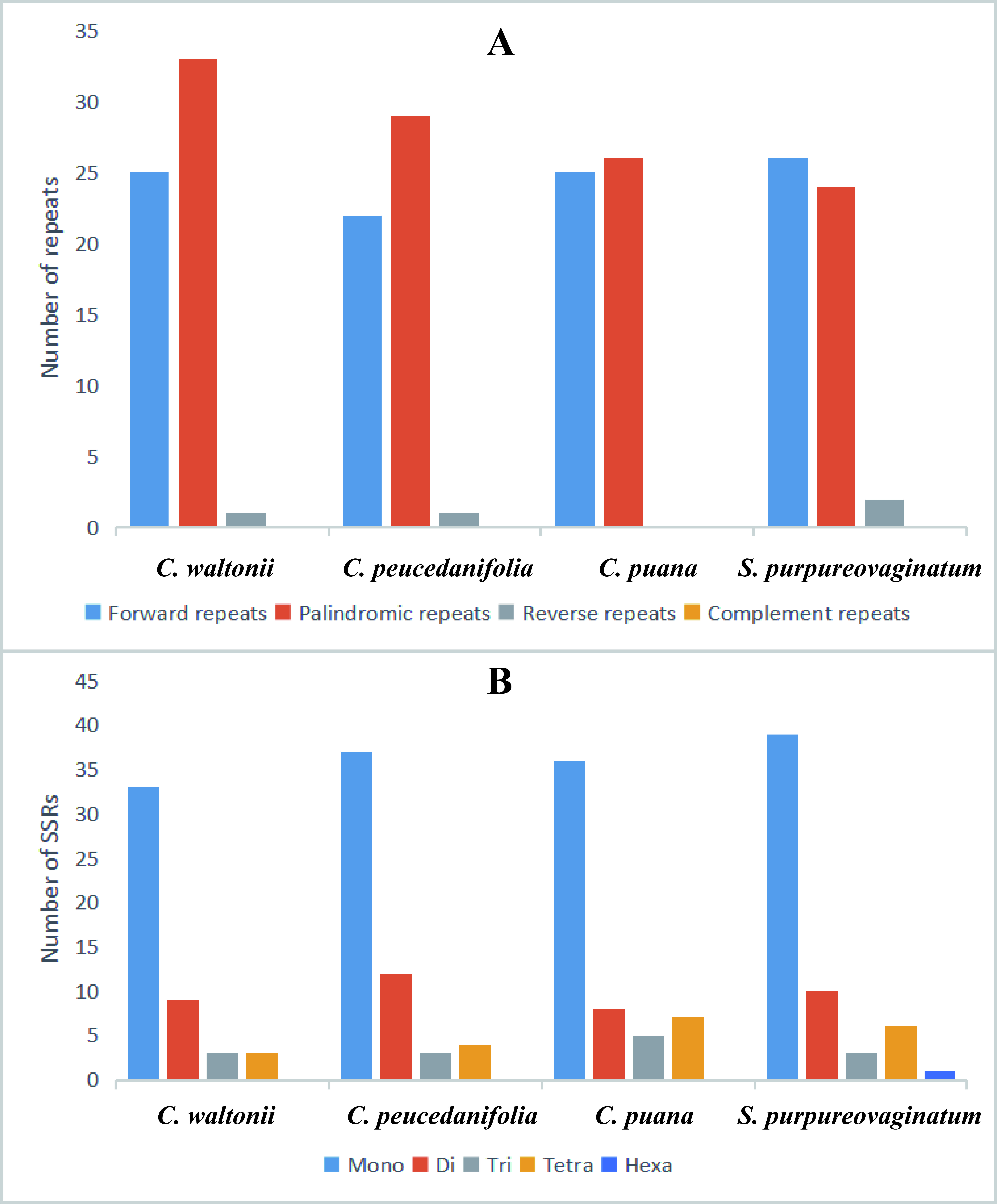

Supplement: Supplementary Figure 3 — Analyses of repeats in the four plastomes. (A) Total number of four repeat types; (B) Total number of SSRs. [file Image3.jpeg]

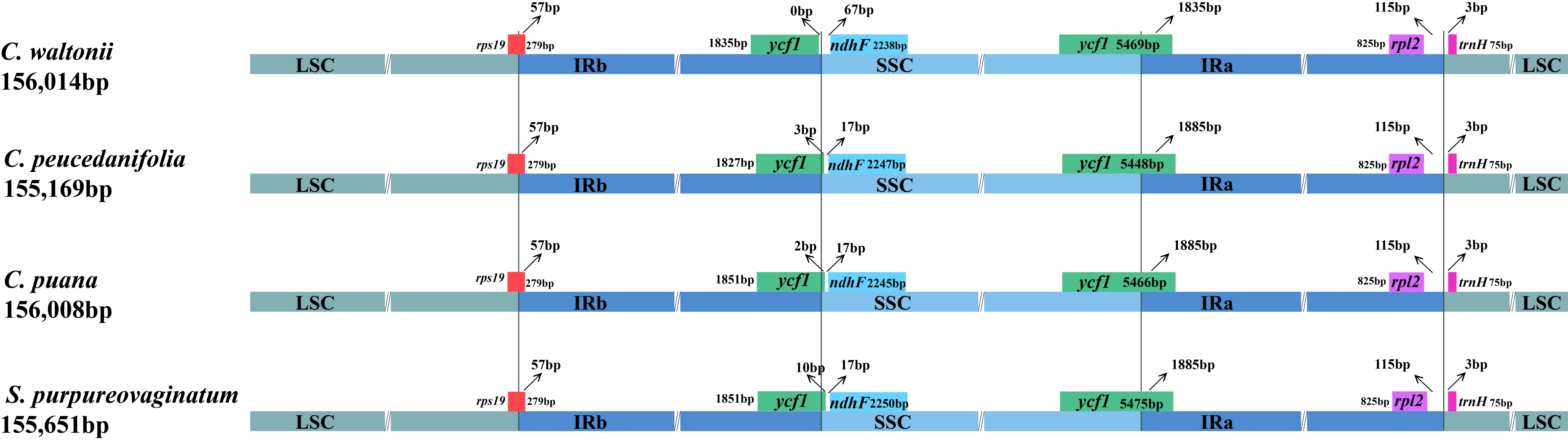

Supplement: Supplementary Figure 4 — Comparison of IR boundaries among four plastomes. [file Image4.jpeg]

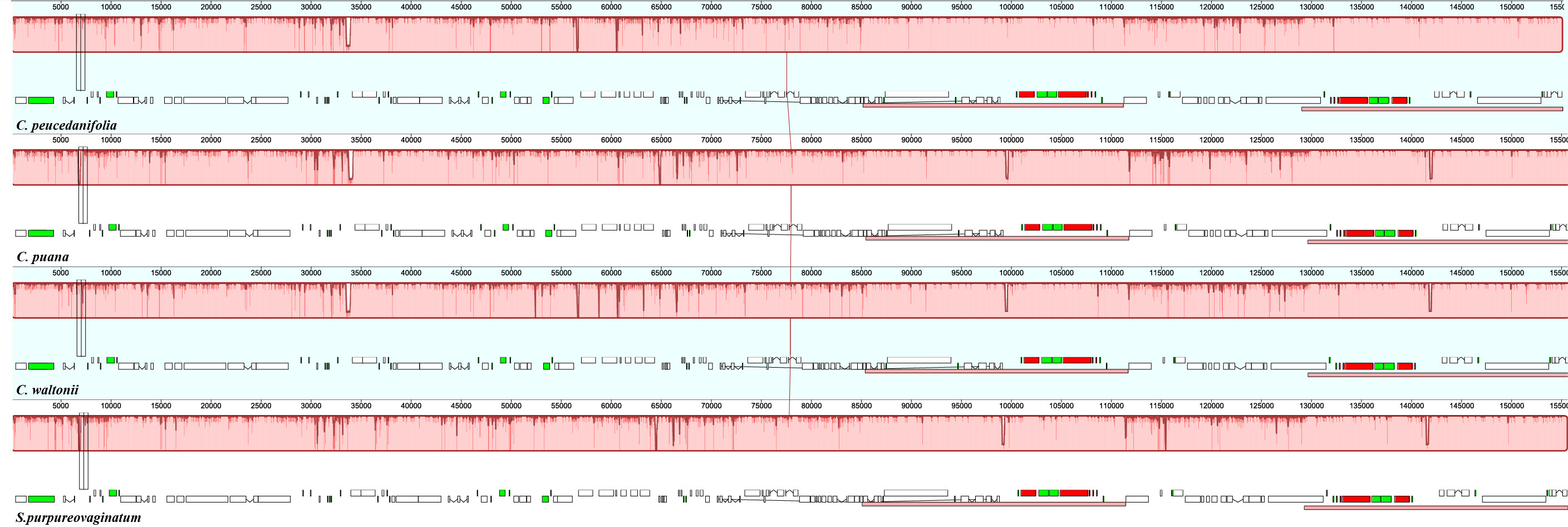

Supplement: Supplementary Figure 6 — Mauve alignment of four plastomes. Local collinear blocks within each alignment are represented by blocks of the same color connected with lines. [file Image6.jpeg]

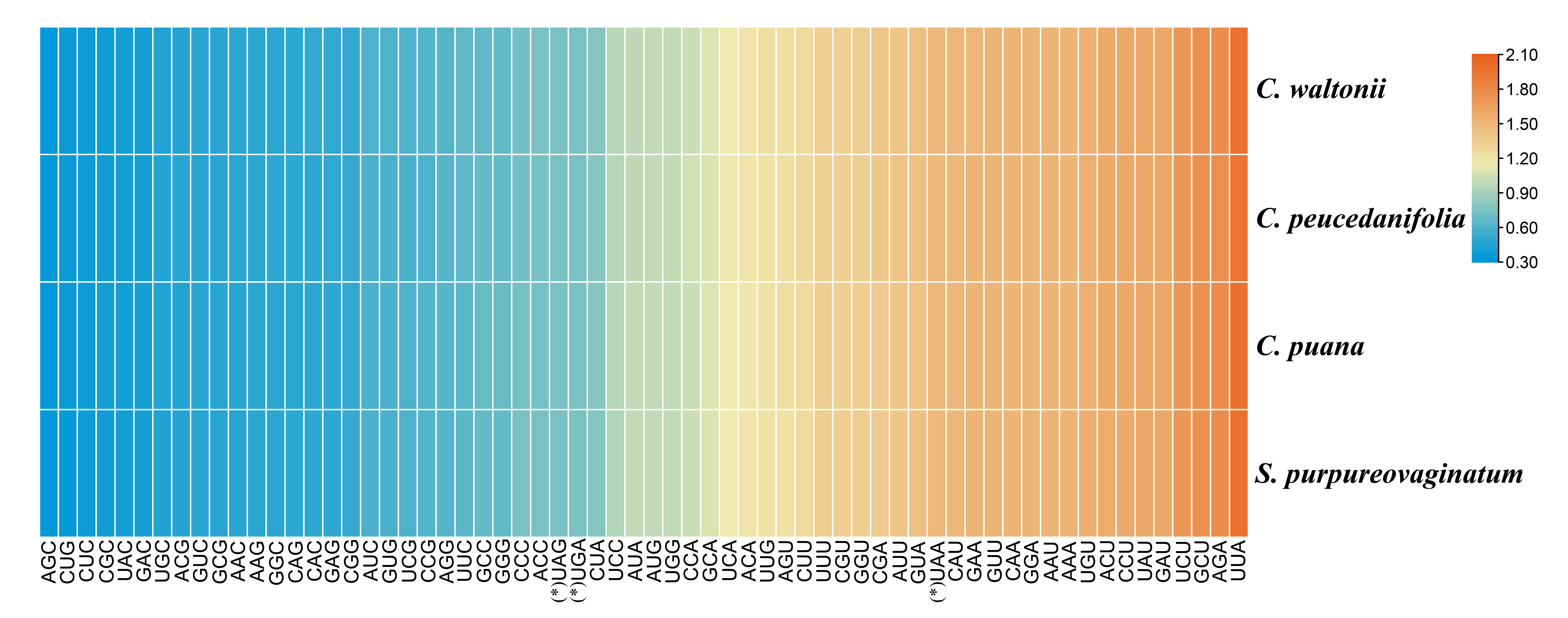

Supplement: Supplementary Figure 7 — The RSCU values of all concatenated protein-coding genes for four plastomes. Color key: higher RSCU values were denoted in red, while lower RSCU values were indicated in blue. * represented the terminator codons. [file Image7.jpeg]
