# Supplementary material for: Morphology, phylogeography, phylogeny, and taxonomy of Cyclorhiza (Apiaceae)
Source: Front Plant Sci. 2025 Jan 8;15:1504734. doi: 10.3389/fpls.2024.1504734 (PMC11750748; doi:10.3389/fpls.2024.1504734)
Supplement: Supplementary file 8 [file Table1.docx]

**Table S1. Sampling information involved in this study.**

| **Population Code** | **Location** | **Latitude (N)** | **Longitude (E)** | **Altitude (m)** | **Sample** | **Voucher Specimen** |
| --- | --- | --- | --- | --- | --- | --- |
| *C. waltonii* |  |  |  |  |  |  |
| SM | Songmai Town, Derong, Ganzi, Sichuan | 28.711622 | 99.288933 | 2375 | 10 | A2022071201 |
| LT | Seweng Village, Litang, Sichuan | 29.04473704 | 100.6109248 | 3253 | 10 | A2022071405 |
| BGT | Binggutuan Village, Daocheng, Sichuan | 29.051339 | 100.461025 | 3609 | 10 | A2022071401 |
| RH | Rihuo Village, Daocheng, Sichuan | 29.056231 | 100.576272 | 3338 | 10 | A2022071403 |
| KD | Shade Town, Kangding, Sichuan | 29.625797 | 101.372850 | 3167 | 8 | A2021101501 |
| AR | Kaga Town, Rikazei, Xizang | 29.30121032 | 87.23233394 | 4344 | 10 | A2023082601 |
| KM | Shaogang Town, Rikazei, Xizang | 28.72664274 | 89.65281218 | 4135 | 10 | A2023082501 |
| JZ | Jiangzi, Rikazei, Xizang | 28.83528074 | 89.86332751 | 4286 | 10 | A2023082201 |
| LS | Benbare, Lasa, Xizang | 29.63675837 | 91.15179317 | 3670 | 10 | A2023081701 |
| ML | Sebidanga Village, Linzhi, Xizang | 29.16989985 | 93.9230043 | 3017 | 10 | A2023081901 |
| BY | Bayi Town, Linzhi, Xizang | 29.7529354 | 94.2270358 | 3068 | 10 | A2023082001 |
| *C. peucedanifolia* |  |  |  |  |  |  |
| LX | Shenmu Village, Linzhi, Xizang | 29.102839 | 93.090872 | 3209 | 10 | G1809290301 |
| GBJD | Mila Mts., Linzhi, Xizang | 29.835733 | 92.324339 | 4814 | 5 | A2022080501 |
| LZ | Renmu Village, Lasa, Xizang | 30.20473861 | 91.3639906 | 4114 | 5 | LZ18100201 |
| MZGK | Jiangzizei Mts., Mozhugongka, Xizang | 30.001297 | 91.905225 | 4377 | 5 | CJ202108100103 |
| RKZ | Zhaxizong Town, Rikazei,Xizang | 28.348472 | 87.198556 | 3947 | 5 | CJ2022080403 |
| JC | Qingping, Jianchuan, Yunnan | 26.55747563 | 99.98450533 | 2631 | 10 | A2023081001 |
| JD | Jiangdong Village, Diqing, Yunnan | 27.95038426 | 99.41539971 | 1996 | 10 | A2022081301 |
| HTX | Hutiaoxia, Diqing, Yunnan | 27.308197 | 100.228378 | 1830 | 5 | CJ202210040301 |
| CS | Shuizhong Village, Lijiang, Yunnan | 26.851178 | 100.185472 | 2479 | 9 | G19102204 |
| LJ | Xiang Mts., Lijiang, Yunana | 26.884497 | 100.237447 | 2454 | 10 | A2022080801 |
| *C. puana* |  |  |  |  |  |  |
| NB | Niba Town, Luohuo, Sichuan | 31.518114 | 100.693386 | 3191 | 10 | A2022070601 |
| RD | Renda, Luohuo, Sichuan | 31.208564 | 100.845239 | 3087 | 8 | A2022101101 |
| NT | Nito Village, Xinglong, Sichuan | 31.181497 | 100.304472 | 3068 | 10 | A2022070901 |
| GS | Gaoshan Village, Xinglong, Sichuan | 30.943817 | 100.313178 | 3080 | 10 | A2022070903 |
| ZK | Zhake Town, Ganzi, Sichuan | 31.798917 | 99.711575 | 3525 | 10 | A2022070801 |
| *S. purpureovaginatum* |  |  |  |  |  |  |
| BR | Baiga Town, Biru, Xizang | 31.19583007 | 94.04366582 | 4011 | 10 | A2023082901 |
