# Supplementary material for: Morphology, phylogeography, phylogeny, and taxonomy of Cyclorhiza (Apiaceae)
Source: Front Plant Sci. 2025 Jan 8;15:1504734. doi: 10.3389/fpls.2024.1504734 (PMC11750748; doi:10.3389/fpls.2024.1504734)
Supplement: Supplementary file 9 [file Table2.docx]

**Table S2. The sequence primers and PCR amplification systems.**

| **cpDNA fragments** | **Primers Sequences (5' – 3')** | **Reference** |
| --- | --- | --- |
| *trn*Q*-rps*16 | F-CCCGCTATTCGGAGGTTCGA  R-ATCGTGTCCTTCAAGTCGCA | Shaw et al.,2007 |
| *trn*D-*trn*T | 5'-ACCAATTGAACTACAATCCC-3'  5'-TTCTTGATCCCTCTTCTCTT-3' | unpublished |
| *mat*K | 5'-GCACTTGATAGATAGCCCAG-3'  5'-TGAAACGTGCAATTGAGCGA-3' | RBG Edinburgh  （unpublished） |
| *rpl*16 | 5'–GCTATGCTTAGTGTGTGACTCGTTG-3'  5'–CCCTTCATTCTTCCTCTATGTTG-3' | Gong et al., 2011 |
| *rpl*32-*trn*L | 5'–GACTTCCAAAAAAACGTACTTC-3'  5'–CTGCTTCCTAAGAGCAGCGT3' | Shaw et al.,2007 |
| *psb*A-*trn*H | 5'-ACTGCCTTGATCCACTTGGC-3'  5'-CGAAGCTCCATCTACAAATGG-3' | Hamilton,1999 |
| *trn*S-*trn*G | 5'-GCCGCTTTAGTCCACTCAGC-3‘  5'-GAACGAATCACACTTTTACCAC-3‘ | Hamilton, 1999 |
| *rbc*L | 5'-ATGTCACCACAAACAGAAAC-3'  5'-TCGCATGTACCTGCAGTAGC-3' | Fay et al. 1997 |
| *rps*16-*trn*K | 5'-TTCCTTGAAAAGGGCGCTCA-3'  5'-TACTCTACCGTTGAGTTAGC-3' | Shaw et al., 2007 |
| *rps*16 | 5'-GTGGTAGAAAGCAACGTGCGACTT-3'  5'-TCGGGATCGAACATCAATTGCAAC-3' | Oxelman et al.,1997 |
