# Supplementary material for: Morphology, phylogeography, phylogeny, and taxonomy of Cyclorhiza (Apiaceae)
Source: Front Plant Sci. 2025 Jan 8;15:1504734. doi: 10.3389/fpls.2024.1504734 (PMC11750748; doi:10.3389/fpls.2024.1504734)
Supplement: Supplementary file 16 [file Table9.docx]

| Population Code | ITS Haplotype (N) | *H*d（SD) | π | Sample |
| --- | --- | --- | --- | --- |
| *C.waltonii* |  |  |  |  |
| SM | N19(10) | 0 | 0 | 10 |
| LT | N20(10) | 0.200 | 0.00033 | 10 |
| BGT | N20(11) | 0 | 0 | 11 |
| RH | N20(10) | 0 | 0 | 10 |
| KD | N20(8) | 0 | 0 | 8 |
| AR | N7(1),N8(1),N13(2),N14(1),N15(2),N17(1) | 0.929 | 0.00923 | 8 |
| KM | N13(1),N14(1),N15(2),N16(4),N17(1),N18(1) | 0.844 | 0.00511 | 10 |
| JZ | N13(3),N14(4),N16(1),N18(1) | 0.750 | 0.00449 | 9 |
| LS | N9(1),N10(5),N13(2),N14(1),N17(1) | 0.756 | 0.01044 | 10 |
| ML | N7(9),N8(1),N14(2) | 0.410 | 0.00553 | 12 |
| BY | N9(3),N10(2),N13(1),N14(3),N16(2) | 0.855 | 0.01030 | 11 |
| DR | N14(1),N16(5) | 0.333 | 0.00278 | 6 |
| LZ | N13(4),N14(1) | 0.400 | 0.00067 | 5 |
| MZGK | N14(5) | 0 | 0 | 5 |
| LX | N11(5),N12(5) | 0.556 | 0.00093 | 10 |
| total |  | 0.867 | 0.02090 | 135 |
| *C. peucedanifolia* |  |  |  |  |
| GBJD | N6(5) | 0 | 0 | 5 |
| JC | N2(12) | 0 | 0 | 12 |
| JD | N1(6),N2(1),N3(3) | 0.600 | 0.00167 | 10 |
| HTX | N5(5) | 0 | 0 | 5 |
| CS | N1(3),N4(6) | 0.500 | 0.00250 | 9 |
| LJ | N1(3),N2(5),N4(2) | 0.689 | 0.00196 | 10 |
| total |  | 0.788 | 0.00354 | 51 |
| *C.puana* |  |  |  |  |
| NB | N24(11) | 0 | 0 | 11 |
| RD | N25(6),N26(2) | 0.429 | 0.00143 | 8 |
| NT | N21(9),N22(1) | 0.200 | 0.00033 | 10 |
| GS | N21(9),N22(1) | 0.200 | 0.00033 | 10 |
| ZK | N23(10) | 0 | 0 | 10 |
| total |  | 0.770 | 0.00525 | 49 |
| 1. *purpureovaginatum* |  |  |  |  |
| BR | N27(4),N28(5) | 0.556 | 0.00093 |  |
| total |  |  |  | 9 |
| all population |  | 0.941 | 0.03051 | 244 |

**Table S9. The haplotype distribution, haplotype diversity (*H*d ) and nucleotide diversity (π) based on ITS sequence.**
