# Supplementary material for: Morphology, phylogeography, phylogeny, and taxonomy of Cyclorhiza (Apiaceae)
Source: Front Plant Sci. 2025 Jan 8;15:1504734. doi: 10.3389/fpls.2024.1504734 (PMC11750748; doi:10.3389/fpls.2024.1504734)
Supplement: Supplementary file 17 [file Table10.docx]

**Table S10. The haplotype distribution, haplotype diversity (*H*d ) and nucleotide diversity (π) based on cpDNA fragments.**

| **Population Code** | **Haplotype (C)** | ***H*d（SD)** | **Pi** | **Sample** |
| --- | --- | --- | --- | --- |
| *C.waltonii* |  |  |  |  |
| SM | C10(10) | 0 | 0 | 10 |
| LT | C10(10) | 0 | 0 | 10 |
| BGT | C10(5),C11(3),C12(1) | 0.536 | 0.00051 | 9 |
| RH | C10(5),C12(3) | 0.536 | 0.00076 | 8 |
| KD | C10(5) | 0 | 0 | 5 |
| AR | C3(1),C5(2),C6(4),C7(1) | 0.750 | 0.00147 | 8 |
| KM | C3(6),C5(2),C6(2) | 0.622 | 0.00084 | 10 |
| JZ | C6(3),C8(6) | 0.500 | 0.00095 | 9 |
| LS | C3(3),C6(7) | 0.467 | 0.00044 | 10 |
| ML | C3(4),C5(4),C6(4) | 0.727 | 0.00115 | 12 |
| BY | C3(3),C6(7),C7(1) | 0.564 | 0.00067 | 11 |
| DR | C10(5) | 0 | 0 | 5 |
| LZ | C1(3),C2(2) | 0.600 | 0.00057 | 5 |
| MZGK | C2(1),C3(3) | 0.500 | 0.00024 | 4 |
| LX | C4(4),C9(3) | 0.571 | 0.00189 | 7 |
| total |  | 0.815 | 0.00379 | 123 |
| *C. peucedanifolia* |  |  |  |  |
| GBJD | C21(4) | 0 | 0 | 4 |
| JC | C15(6),C16(6) | 0.545 | 0.00052 | 12 |
| JD | C13(2),C18(6),C19(4) | 0.667 | 0.00166 | 12 |
| HTX | C20(5),C21(1) | 0.333 | 0.00047 | 6 |
| CS | C13(2),C14(2),C17(4) | 0.714 | 0.00074 | 8 |
| LJ | C13(1),C17(11) | 0.167 | 0.00024 | 12 |
| total |  | 0.869 | 0.00391 | 54 |
| *C.puana* |  |  |  |  |
| NB | C23(2),C24(2),C25(1),C32(1) | 0.867 | 0.00198 | 6 |
| RD | C22(3),C24(1),C25(2),C33(2) | 0.821 | 0.00354 | 8 |
| NT | C29(4),C31(2) | 0.533 | 0.00328 | 6 |
| GS | C27(2),C28(2),C29(1),C30(2) | 0.867 | 0.00249 | 7 |
| ZK | C22(2),C24(1),C26(7) | 0.511 | 0.00041 | 10 |
| total |  | 0.916 | 0.00289 | 37 |
| *S.purpureovaginatum* |  |  |  |  |
| BR | C34(2),C35(5) | 0.476 | 0.00045 | 7 |
| all population |  | 0.9324 | 0.00927 |  |
